# Patient and provider experiences with active surveillance: A scoping review

**DOI:** 10.1371/journal.pone.0192097

**Published:** 2018-02-05

**Authors:** Claire Kim, Frances C. Wright, Nicole J. Look Hong, Gary Groot, Lucy Helyer, Pamela Meiers, May Lynn Quan, Robin Urquhart, Rebecca Warburton, Anna R. Gagliardi

**Affiliations:** 1 Department of Clinical Decision Making & Health Care, University Health Network, Toronto, Ontario, Canada; 2 Department of Surgery, Sunnybrook Health Sciences Centre, Toronto, Ontario, Canada; 3 Division of Surgical Oncology, Sunnybrook Health Sciences Centre, Toronto, Ontario, Canada; 4 Department of Medicine, University of Saskatchewan, Saskatoon, Saskatchewan, Canada; 5 Division of General Surgery, Dalhousie University, Halifax, Nova Scotia, Canada; 6 Department of Surgery, University of Saskatchewan, Saskatoon, Saskatchewan, Canada; 7 Department of Oncology, University of Calgary, Calgary, Alberta, Canada; 8 Department of Surgery, Dalhousie University, Halifax, Nova Scotia, Canada; 9 Department of Surgery, University of British Columbia, Vancouver, British Columbia, Canada; 10 Department of Clinical Decision Making & Health Care, University Health Network, Toronto, Ontario, Canada; Carolina Urologic Research Center, UNITED STATES

## Abstract

**Objective:**

Active surveillance (AS) represents a fundamental shift in managing select cancer patients that initiates treatment only upon disease progression to avoid overtreatment. Given uncertain outcomes, patient engagement could support decision-making about AS. Little is known about how to optimize patient engagement for AS decision-making. This scoping review aimed to characterize research on patient and provider communication about AS, and associated determinants and outcomes.

**Methods:**

MEDLINE, EMBASE, CINAHL, and The Cochrane Library were searched from 2006 to October 2016. English language studies that evaluated cancer patient or provider AS views, experiences or behavioural interventions were eligible. Screening and data extraction were done in duplicate. Summary statistics were used to describe study characteristics and findings.

**Results:**

A total of 2,078 studies were identified, 1,587 were unique, and 1,243 were excluded based on titles/abstracts. Among 344 full-text articles, 73 studies were eligible: 2 ductal carcinoma in situ (DCIS), 4 chronic lymphocytic leukemia (CLL), 6 renal cell carcinoma (RCC) and 61 prostate cancer. The most influential determinant of initiating AS was physician recommendation. Others included higher socioeconomic status, smaller tumor size, comorbid disease, older age, and preference to avoid adverse treatment effects. AS patients desired more information about AS and reassurance about future treatment options, involvement in decision-making and assessment of illness uncertainty and supportive care needs during follow-up. Only three studies of prostate cancer evaluated interventions to improve AS communication or experience.

**Conclusions:**

This study revealed a paucity of research on AS communication for DCIS, RCC and CLL, but generated insight on how to optimize AS discussions in the context of routine care or clinical trials from research on AS for prostate cancer. Further research is needed on AS for patients with DCIS, RCC and CLL, and to evaluate interventions aimed at patients and/or providers to improve AS communication, experience and associated outcomes.

## Background

Active surveillance (AS) is a relatively new option for managing select cancer patients to reduce over-treatment and the associated sequelae that can impact health and health-related quality of life [1]. AS avoids or postpones definitive cancer treatment until there is evidence from periodic observation or testing that a patient is at risk of or has disease progression [2]. AS is considered a management option for patients with prostate cancer [3,4], and research is accumulating to establish the clinical effectiveness of AS for renal cell carcinoma (RCC) [5,6], ductal carcinoma in situ (DCIS) of the breast [7], follicular lymphoma [8] and chronic lymphocytic leukemia (CLL) [9].

Patients are known to vary in their personal preferences for level of acceptable risk associated with disease management [10]. Some patients who chose AS reported greater health-related quality of life compared with those who had treatment [1] while others reported experiencing fear and anxiety about disease progression [11,12]. Thus it is important to fully inform cancer patients who are potentially eligible for AS about management options and associated implications, and provide the opportunity, if they desire, to take part in treatment decisions. Patient engagement (PE) [13] in their own care is particularly relevant in circumstances where there is limited evidence to support decision-making, two or more treatment options are suitable, or treatment outcomes are difficult to predict or may be adverse, as is the case for many types of cancer (20). Such treatment decisions are considered “preference-sensitive” because patients fully informed of the risks and benefits might change their treatment preference [14]. PE improves numerous patient (i.e. experience, satisfaction) and health system outcomes (i.e. cost-effective service delivery and use) [14–16], but is more probable if strategies to support it are aimed not only at patients, but also at physicians who influence treatment choices [17].

AS represents a fundamental shift in patient care that may cause patient and provider uncertainty. However, most AS research has focused on establishing the effectiveness of AS compared with other treatment options, the clinical indications for selecting patients eligible for AS, and optimal clinical procedures for monitoring cancer progression [18,19]. Hence, insight is needed on patient or provider views and experiences of AS to identify behavioural interventions that may be needed by one or both groups to be aware of, understand, and engage in discussions about the implications of AS. The purpose of this study was to describe the characteristics and findings of research that evaluated patient or physician views and experiences of AS, and the characteristics of behavioural interventions that have been used to promote or support PE in AS discussions. Overall, the findings could reveal gaps in knowledge to guide ongoing research, detail uncertainties and concerns among patients and physicians about AS and the support they need for PE, and identify interventions that could provide needed PE support for AS discussions. Ultimately, by contributing to widespread implementation of PE in decisions about AS, knowledge generated by this research could optimize the clinical and psychosocial sequelae of AS, reduce undue harm to patients, and improve patient, provider and health system outcomes.

## Materials and methods

### Approach

Given that the clinical effectiveness of individual treatments was not addressed, a traditional systematic review was not conducted. Instead, a scoping review was chosen as the methodologic approach [14–16]. Similar in rigour to a systematic review, the purpose of a scoping review is to gain an understanding of the extent of research on a given topic, reveal gaps in knowledge, and identify issues warranting ongoing research [20]. A scoping review involves five steps: scoping the literature, searching, screening, data extraction and data analysis. The Preferred Reporting Items for Systematic Reviews and Meta-Analyses [21] criteria guided reporting of the methods and findings [14]. Data were publicly available so institutional review board approval was not needed. A protocol for this review was not registered.

### Scoping

The scoping process involved becoming familiar with the literature on this topic. A preliminary search was conducted in MEDLINE using Medical Subject Headings including, but not limited to [watchful waiting or active surveillance] and [patient education as topic or patient-centered care]. The term watching waiting (WW) was included because it is used interchangeably with active surveillance as it also involves monitoring or observation until treatment of disease or symptoms is warranted, and may capture relevant studies about patient or provider views and experiences of delayed treatment not labelled as AS. [12] CK and ARG screened titles and abstracts of the preliminary search results, which were used to plan a more comprehensive search strategy and to generate eligibility criteria based on the PICO (population, intervention, comparisons, outcomes) framework. All members of the research team, comprised of health services researchers and general surgeons who care for cancer patients, reviewed eligibility criteria and provided feedback.

Populations referred to both patients and physicians. Patients included those diagnosed with prostate cancer, DCIS, RCC, or CLL, which are types of cancer for which AS has been used [14–16]. It is likely that more studies of AS will involve prostate cancer because the effectiveness of AS for other types of cancer has not yet been definitively established; however, much could be learned about PE in AS discussions from prostate cancer studies that could potentially inform practice or research for other types of cancer. Providers were practicing physicians who manage patients with those conditions including general surgeons, and surgical, radiation and medical oncologists. Although other types of clinicians may interact with these patients (i.e. nurse navigators), physicians are more likely to influence treatment choices [17].

Interventions included clinical and behavioural interventions. The clinical intervention was labelled as AS, WW or another synonymous term such as observation, provided it referred to periodic follow-up or testing of patients for signs of cancer progression or symptoms that warrant treatment [1]. Behavioural interventions included any policy, program or single or multi-faceted strategy implemented to promote awareness, understanding and discussion about AS/WW.

With respect to comparisons, studies were eligible if they explored or evaluated the following aspects of AS/WW: understanding of its purpose and processes; views about its purpose and value; communication about AS; experiences and psychosocial outcomes of undergoing AS/WW; determinants or factors influencing AS/WW understanding, views, communication, experiences or choice; or behavioural interventions to support or improve any of these functions by comparing patients or providers with and without exposure to interventions, or before or after exposure to interventions, or receiving different types of interventions.

Outcomes were those reported in eligible studies, and included but were not limited to awareness, understanding, communication, experiences or impact of AS/WW, or determinants or factors influencing any of these functions, or the impact of behavioural interventions implemented to support or improve any of these functions. Eligible study designs included English language qualitative (interviews, focus groups, qualitative case studies), quantitative (questionnaires, randomized controlled trials, time series, before/after studies, prospective or retrospective cohort studies, case control studies) or mixed methods studies. Systematic reviews were not eligible but their references and those of all eligible studies were screened to identify additional eligible primary studies.

### Searching

The search strategy was developed in conjunction with a medical librarian and complied with the Peer Review of Electronic Search Strategy reporting guidelines (S1 File) [22]. MEDLINE, EMBASE, CINAHL, and the Cochrane Library were searched on November 25, 2016 from 2006 to that date. The year 2006 was chosen to capture the most recent ten years of research Although AS is a relatively new phenomenon, the year 2006 was chosen to capture studies about delayed treatment potentially referred to as watchful waiting.

### Screening

To prepare for screening, CK and ARG independently screened the title and abstract of the first 25 search results, then compared and discussed discrepancies and how to interpret and apply the eligibility criteria. CK and ARG screened titles and abstracts according to specified PICO-based eligibility criteria. Criteria for ineligible studies were generated prospectively with screening. Studies were not eligible if they involved health care providers other than physicians, nurses or allied health care professionals (including but not limited to physiotherapists, speech therapists, occupational therapists, social workers, pharmacists, etc.); involved trainee physicians such as interns, residents or fellows (studies were included if at least half of the participants were practicing physicians); examined the effectiveness or impact of AS compared with active treatment; focused on watchful waiting not in the context of cure but as an option for frail or elderly patients not eligible for treatment; or were in the form of protocols, editorials, commentaries, letters, news items, meeting abstracts or proceedings. All items selected by at least one reviewer were retrieved.

### Data extraction

A data extraction form was developed to collect information on study characteristics including author, publication year, country and type of cancer, study objective, research design, participants, focus on AS or WW, and findings. If a behavioural intervention was employed, data were also extracted on content (information/knowledge conveyed), format (mode of delivery, single or multi-faceted), timing (duration, frequency), participants (number, type, setting) and personnel who delivered the intervention according to the Workgroup for Intervention Development and Evaluation Research [23] reporting standards for behavioural interventions [14–16]. To pilot data extraction, CK and ARG independently extracted data from the same three articles, and compared and discussed findings to refine the data extraction form. CK extracted data from all articles, which were independently checked by ARG.

### Data analysis

Summary statistics were used to report the number of studies published per year, by type of cancer, in different countries and according to study design. Study findings were tabulated and reported narratively by type of cancer. Methodological quality of included studies was not assessed as this is not customary for a scoping review.

## Results

### Search results

**Fig 1:**
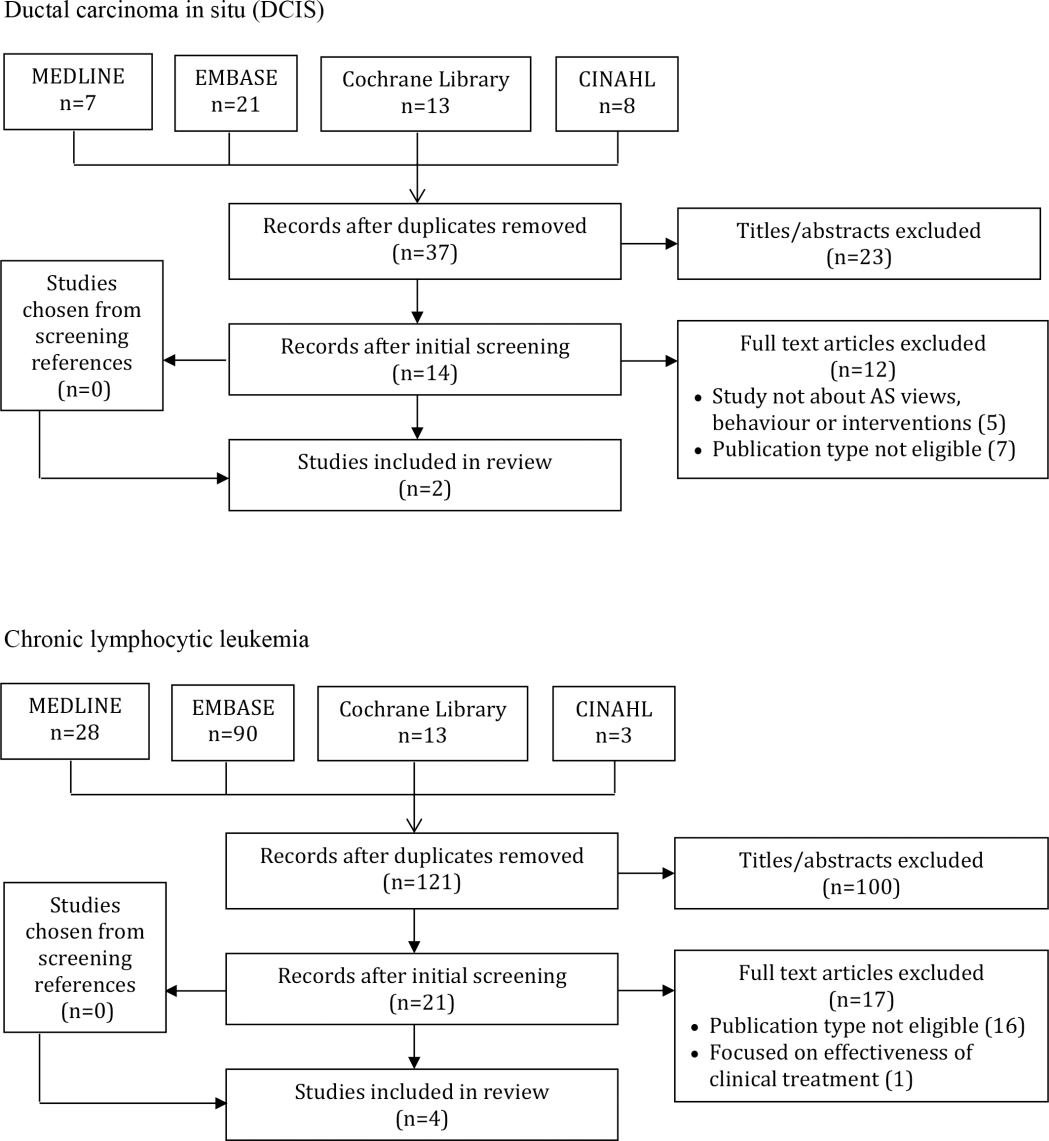
PRISMA diagrams.

A total of 2,078 studies were identified by searches, of which 1,587 were unique items, and 1,243 were excluded based on screening of titles and abstracts. Among 344 full-text articles that were screened, 269 were excluded because the publication type was not eligible (138), studies did not assess AS/WW views, behaviour or interventions (110), focused on effectiveness of clinical treatment (12), studies were preliminary or ongoing (8), not English language (4), or duplicate (1). Of 516 systematic reviews identified through screening, three were relevant and two additional eligible primary studies were identified among their references. A total of 73 studies were eligible for review including 2 DCIS, 4 CLL, 6 RCC and 61 prostate cancer studies (Fig 1). Data extracted from included studies are available in S1 Table [24–96].

### Study characteristics

**Fig 2:**
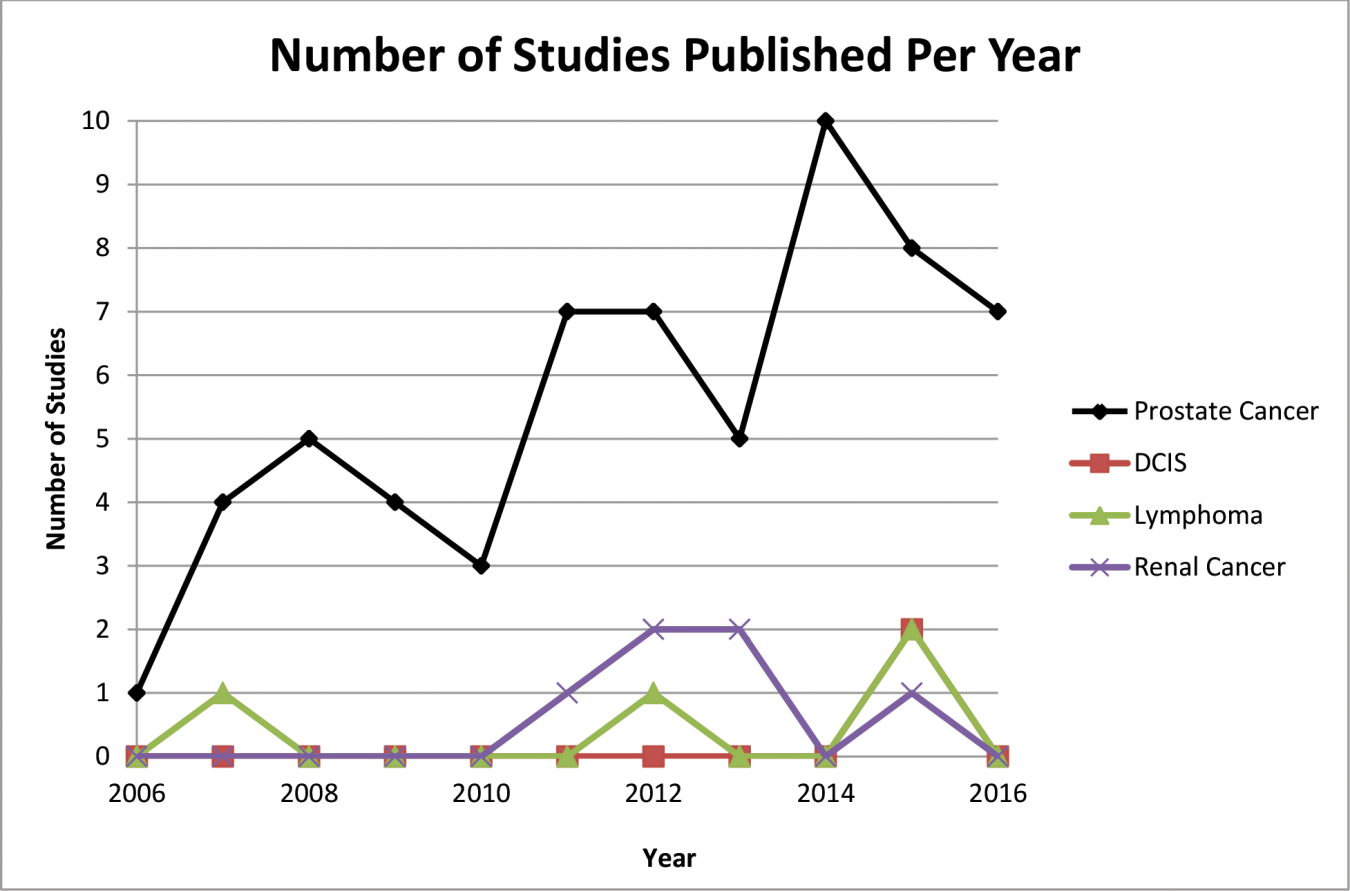
Studies published per year.

The number of studies published per year increased almost annually for prostate cancer from 2006 to 2016; studies pertaining to DCIS, RCC and CLL were largely published between 2012 and 2016 (Fig 2). Studies were conducted in the United States (39), Netherlands (8), Canada (8), Australia (7), United Kingdom (6), Belgium (1), Denmark (1), Finland (1), Norway (1) and Switzerland (1). The majority of included studies involved prostate cancer (61, 83.6%) followed by RCC (6, 8.2%), CLL (4, 5.5%) and DCIS (2, 2.7%). With respect to research design, most studies involved single cohorts (27, 37%) followed by comparative cohorts (18, 24.6%), qualitative interviews or focus groups (17, 23.3%), and cross-sectional questionnaires (11, 15.1%).

### DCIS

Two studies exploring the option of AS for DCIS focused on the language used to describe DCIS. In one study of 269 women, those first exposed to the term abnormal cells then later pre-invasive breast cancer cells were more likely to feel concern (p = 0.001) and change their management preference to treatment (p = 0.008) compared to women exposed first to the term pre-invasive breast cancer and then abnormal cells, however, there was no significant difference in treatment preferences between the two groups (p = 0.23) [24]. In the other study, 26 women who were interviewed said that they would feel concern regardless of the term used to describe DCIS but preferred the term abnormal cells over other terms such as carcinoma, and expressed interest in AS provided monitoring was very frequent [25].

### CLL

Three of four studies of AS or WW among CLL patients focused on quality of life; the fourth study examined satisfaction with CLL information and follow-up appointments. Two of three studies that employed cross-sectional questionnaires found that CLL patients undergoing treatment experienced significantly lower quality of life compared with those undergoing WW: emotional and social functioning (p = 0.004) and fatigue (p = 0.021) [26]; or AS: worry about the future (p = 0.02) [27]. The third cross-sectional study found no significant difference in depression, anxiety and physical/mental quality of life among those undergoing treatment or WW (p>0.10), though patients undergoing WW who were aged 60 or younger experienced more depression (p = 0.014) and significantly worse emotional (p = 0.0001) and social quality of life (p = 0.002) compared with those who were older [29]. In one study, 12 CLL patients undergoing WW who were interviewed said they received very little information about their condition and wanted to know more about it and how it might affect them in the future; were dissatisfied with follow-up appointments, which felt cursory; and experienced depression and anxiety [28].

### RCC

Five of six studies of AS among RCC patients focused on determinants of AS; one study examined factors associated with illness uncertainty. Four studies referred to AS, [32–35] one to WW, [31] and one to observation. [30] In a study of 7,047 patients, disease factors including having multiple comorbidities, poor, African American, and uninsured or socially insured patients (OR, 3.45; CI, 2.92 to 4.09 and OR, 1.76; CI 1.62 to 1.91; p<0.01); and those receiving care at community or low-volume hospitals (OR, 1.11; CI, 1.03 to 1.20; p<0.01) were more likely to undergo observation [30]. Among a single-institution cohort of 1034 patients, 266 (25.7%) were managed with AS [32]. The use of AS did not change significantly over time from 2005 to 2010 (12% versus 20%, p = 0.09) and was less likely than treatment with decreasing tumor size (OR 0.2, 95% CI 0.1 to 0.3) and high complexity nephrectomy (OR 0.1, 95% CI 0.03 to 0.3). Among 26,468 patients with RCC identified in the Surveillance, Epidemiology and End Results database, 2,797 (11.0%) underwent AS [33]. The rate of AS increased over time from 2.4% in 1988 to 18.2% in 2008 (p<0.001) and AS was associated with more contemporary year of diagnosis, older age, male gender, decreasing tumor size and unmarried marital status (p≤0.001). In a single-institution cohort of 204 patients, 73 (36.0%) underwent AS [34]. Tumor size less than 3 cm, Eastern Cooperative Oncology Group performance status of ≥2 and an endophytic lesion were most predictive of AS, and surgeons using primarily an open surgical approach were more likely to choose AS compared with those using a robotic approach (OR 4.5, 95% CI 0.61 to 3.44). A questionnaire survey of 759 American Urological Association members found that respondents were more likely to choose AS for patients who were older (OR 2.7, 95% CI 2.1 to 3.6, p<0.0001), had comorbidities (OR 10.0, 95% CI 8.0 to 12.4, p<0.0001), and with smaller tumor size (OR 0.18, 95% CI 0.15 to 0.21, p<0.0001) or perihilar (OR 2.0, 95% CI 1.8 to 2.3, p<0.001) or polar tumor (OR 2.1, 95% CI 1.9 to 2.5, p<0.0001) [35]. Among 100 patients on WW from a single cancer centre who responded to a questionnaire survey, greater illness uncertainty was associated with poorer general quality of life scores in the physical domain (p = 0.008); worse cancer-related quality of life in physical (p = 0.001), psychosocial (p <0.001) and medical (p = 0.034) domains, and higher distress (p <0.001) [31].

### Prostate cancer

A total of 61 studies examined AS or WW for prostate cancer; 50 studies referred to AS (82%) and a few referred to WW [51,75,84,87–90,93,95,96] or used AS and WW interchangeably. [66]

#### Information, communication and decision-making

Fourteen (23.0%) of 61 studies examined information or communication needs among AS patients. Three studies found that patients had limited knowledge about AS [52,86] or WW. [66] Those who felt they had not received sufficient information reported high rates of stress [96]. A study of normative messaging found that men needed reassurance that AS is likely to allow time for curative treatment if the cancer progresses [48]. In two studies, men wished to be discrete about their condition, did not discuss concerns with their spouse and/or other support group structures, and used the Internet as a primary source of information about prostate cancer [45,74]. In another study, men on AS said they felt neglected due to limited time with providers and experienced difficulty finding information about AS [80]. In a study involving interviews with men on AS, some reported brief discussion with physicians and were stoic in accepting physician recommendations for AS, while others were confused and anxious about their diagnosis and the AS process, lost or lacked confidence in their physician, and desired a more collaborative decision-making process [64]. Three additional studies found that AS patients preferred informed decision-making and playing an active role with 41% of men assuming a shared role [38,72,76]. Individuals with higher self-efficacy for prostate cancer symptom management and higher positive meaning for cancer were less likely to express decision-making conflict [68]. In two studies, female partners were said to play a considerable supportive role in the decision-making process [52,86]. Another study found that men undergoing AS in the United States had slightly higher levels of decisional uncertainty compared with men in Ireland [91].

#### Determinants of AS

Twenty-two (36.1%) of 61 studies examined factors that were associated with AS. Surveys of American physicians revealed that they believed AS to be an effective but underused management strategy [54,70]. American and British urologists who were surveyed said they most commonly used predicted 10-year survival probability, MRI/laparoscopy stage and prostate specific antigen test results, as well as patient characteristics such as age, comorbidities and history of compliance when deciding who was an appropriate AS candidate [39,94]. Patients said that primary factor influencing the decision to choose AS or WW was physician recommendation [36,57,69,72,75,76,78,79,86,89]. Other factors associated with AS or WW included trust in their physician; urologists with oncology fellowships [43] or urologist referral [90]; desire to prolong good health, avoid the side effects of invasive treatment such as incontinence and erectile dysfunction [36,57,75–78,86,89]; older age [38,42,51,59,60,65,76,87,90], more comorbidities [87], higher education [42], distance from home to medical centre [90], greater awareness of having low-risk cancer [42], lower levels of anxiety or depression [61], and preference or opportunity for shared decision-making [42,56].

#### Anxiety, depression and quality of life (QoL)

Twenty-two (36.1%) of 61 studies assessed the psychosocial sequelae of AS. Compared with non-cancer patients, men on AS or WW experienced declined urinary and bowel function and had bodily pain but maintained sexual activity and there was no significant difference in QoL [40,49,58,93]. In contrast, one study found that WW patients had lower QoL compared with men without prostate cancer [87]. Several studies found that patients on AS or WW experienced better general mental and physical health-related QoL, and lower levels of anxiety and dysfunction in work role and daily activities compared with those undergoing radical prostatectomy or radiotherapy [50,53,62,69,71,81–84,92]. QoL was associated with having a partner and a physician who engaged in collaborative decision-making [96,97]. In contrast, some studies reported that AS patients experienced greater anxiety or depression compared with those undergoing treatment [46,62,84,92], which was associated with illness uncertainty/fear of progression [74,94]. Coping mechanisms reported by patients included receiving information about future treatment options, having high coping confidence [47], talking to other men or joining a support group for men on the same program [76], choosing to consider prostate cancer as benign in order to lead a normal life [85]; and keeping busy with work, self-care, alternative medications and prayer [95].

#### Behavioural interventions

Three (4.9%) of 61 studies evaluated the impact of behavioural interventions aimed at patients or providers to promote or support AS or WW. One study evaluated an interactive Internet-based educational module on treatment options and found that patients had greater intention to accept AS after completing the module [73]. One study evaluated a treatment decision aid including the option of WW and found that patients became more active partners in the decision-making process after reviewing the decision aid [88]. One study evaluated a nurse-led AS clinic and found that 30% to 40% of patients in both the intervention group and a comparative group receiving standard urologist follow-up similarly reported health-related distress, worry, feeling low, and insomnia [44].

## Discussion

Evidence for the effectiveness of AS for DCIS, RCC or CLL is accumulating, [5–9] yet this scoping review found a paucity of research on patient or provider views and experiences of PE in decision-making for AS compared with prostate cancer for which AS is a standard option. [3,4] Among patients with DCIS, there was no difference in concern or treatment preferences if the condition was referred to as “abnormal cells” or “cancer”. Despite differences in disease characteristics, there were similarities in determinants and outcomes of AS/WW. The most influential determinant of AS was physician recommendation. Among patients with CLL or prostate cancer, other determinants included higher SES, smaller tumor or comorbid disease, older age, desire to avoid the physiological side effects of treatment, and preference for shared decision-making. Patients with CLL or prostate cancer were dissatisfied with information received about AS and the follow-up process, which they felt was cursory, and desired greater involvement in treatment decision-making. QoL studies with CLL and prostate cancer patients generated mixed findings–in most cases those on AS reported higher QoL compared with those undergoing treatment but a few studies showed that those on AS experienced greater anxiety and depression. This may be related to illness uncertainty–those with RCC and prostate cancer who were unclear about prognosis were more likely to experience anxiety and depression–they said it was important to be reassured about the potential for future treatment options if necessary. Only three studies of prostate cancer evaluated interventions aimed at patients and/or providers to improve AS communication or experience: Internet-based education resulted in greater intention to consider AS, a decision aid resulted in more active involvement in decision-making, and a nurse-led AS clinic resulted in similar rates of anxiety and depression compared with standard urologist follow-up.

This study featured both strengths and limitations. We searched the most relevant databases of medical literature with a search strategy that complied with standards [24], and employed rigorous searching and screening processes. Comparison of the characteristics of research on views and experiences of AS for DCIS, RCC and CLL with prostate cancer, which features a greater volume of research accumulated over the last ten years, generated learning and insight on the type of studies needed for those types of cancer. A few issues may limit the interpretation and use of these findings. We may not have identified all relevant studies. We did not search the grey literature, assuming that most empirical research would be found in indexed databases. Publication bias, or the tendency for journals to publish studies with positive results or surveys with high response rates, may have influenced the number and type of studies that were retrieved. It is difficult to compare findings for different types of cancer due to potentially differing definitions and processes for AS. Due to the small number of studies on DCIS, RCC and CLL, for which the clinical effectiveness of AS remains unclear, findings are uncertain and knowledge about AS views and experiences from the considerably greater number of prostate cancer studies, for which the effectiveness of AS has been established, cannot necessarily be applied to other types of cancer although some similarities were identified. Also, use of AS may vary internationally based on health care system characteristics, contributing to the variable findings of this review. Given the wide range of processes and outcomes measured and reported across included studies it was not possible to pool findings. However, the purpose of this study was to assess the state of research on AS and serve as a springboard for ongoing research in this area.

Several findings suggest avenues for improving the quality of care for patients who may be eligible for AS in the context of routine care or clinical trials. Most patients desired more information about treatment options and prognosis, reassurance that if AS was chosen curative treatment would be possible in the future, and involvement in decision-making. As the primary determinant of AS was physician recommendation, and because QoL for those on AS was similar to the general population and greater than those undergoing treatment, patient experience and outcome could be improved by ensuring that patients are aware of and given the opportunity to discuss AS. PE in their own health care has been associated with improved behavioural, psycho-social and clinical outcomes [14–16], however, research has shown that physicians lack aptitude for PE in general [17]. Two studies explored physician perspectives on DCIS: 22% of 296 physicians in the United Kingdom, and 78% of 151 physicians in the United States said that it was difficult to explain DCIS and treatment options to patients [97,98]. Hence, physicians may require training or knowledge-based tools to better engage patients in discussions about the option of AS.

Patients undergoing AS also said that communication during follow-up was cursory and inadequate. QoL among AS patients was lower for those who lacked coping mechanisms and those with illness uncertainty. In general, cancer patients and survivors experience a wide range of unmet supportive care needs [99,100]. Numerous supportive care interventions are available to help cancer patients or survivors manage common problems such as stress and uncertainty [101,102]. For men with prostate cancer on AS, spousal and social support were important in helping them to cope with their chosen care plan [103]. Thus, follow-up for AS patients could be improved by assessing QoL, and providing or linking patients experiencing stress related to illness uncertainty with various forms of supportive care.

With respect to ongoing research, more investigation is needed on the implications of AS for patients with DCIS, RCC and CLL. Trials are currently underway for DCIS to establish clinical and psychosocial outcomes of AS for DCIS [104,105]. Given the variety of determinants associated with AS expressed by patients and providers, further research is needed to establish clinical criteria for AS that could complement and support shared decision-making for AS. This review identified a paucity of research on interventions aimed to patients and/or providers to support knowledge about and shared decision-making for AS. Therefore studies are needed to develop, implement and evaluate behavioural interventions that could improve the patient and provider experience, and associated process and clinical outcomes.

## Conclusions

AS represents a major shift in cancer management about which patients are providers are uncertain. This scoping review found that patients with DCIS, RCC, CLL or prostate cancer undergoing AS in the context of routine care or clinical trials may require more information about the process and future treatment options, involvement in decision-making and assessment for illness uncertainty and supportive care needs during follow-up. A wide range of determinants were associated with AS, suggesting the need for patient selection criteria that could facilitate shared decision-making. Further research is needed to evaluate interventions aimed at patients and/or providers to improve AS communication, experience and associated outcomes.

## Supporting information

S1 File Search strategy. (DOCX)

S2 File PRISMA checklist. (DOCX)

S1 Table Data extracted from included studies. (DOCX)
