# Supplementary material for: Patient and provider experiences with active surveillance: A scoping review
Source: PLoS One. 2018 Feb 5;13(2):e0192097. doi: 10.1371/journal.pone.0192097 (PMC5798833; doi:10.1371/journal.pone.0192097)
Supplement: S1 File — (DOCX) [file pone.0192097.s001.docx]

Supporting File 1. Search strategy

1     Watchful Waiting/ (2068)
2     active [surveillance.mp](http://surveillance.mp/). (3867)
3     watchful [waiting.mp](http://waiting.mp/). (3416)
4     expectant [management.mp](http://management.mp/). (1480)
5     medical [vigilance.mp](http://vigilance.mp/). (13)
6     medical [surveillance.mp](http://surveillance.mp/). (848)
7     Observation/ (5153)
8     [observation.mp](http://observation.mp/). (149522)
9     conservative [management.mp](http://management.mp/). (7159)
10     active [monitoring.mp](http://monitoring.mp/). (248)
11     "watch and wait".mp. (350)
12     "wait and watch".mp. (98)
13     or/1-12 (165191)
14     decision making/ or choice behavior/ (76475)
15     exp Patient-Centered Care/ (13358)
16     Patient Participation/ (15978)
17     Physician-Patient Relations/ (40325)
18     Health Knowledge, Attitudes, Practice/ (77982)
19     Health Literacy/ (2787)
20     Patient Education as Topic/ (55337)
21     Attitude to Health/ (55521)
22     Practice Patterns, Physicians'/ (43186)
23     communication barriers/ (4573)
24     Health Communication/ (1046)
25     decision support techniques/ or decision support systems, clinical/ (18925)
26     Quality Improvement/ (11454)
27     patient care management/ or disease management/ (19816)
28     quality of health care/ (44625)
29     quality assurance, health care/ (39172)
30     "Quality of Life"/ (126467)
31     "cost of illness"/ (19780)
32     exp Self Care/ (34371)
33     adaptation, psychological/ or emotional adjustment/ (57423)
34     exp Patient Satisfaction/ (65676)
35     or/14-34 (664112)
36     13 and 35 (8771)
37     Carcinoma, Intraductal, Noninfiltrating/ (4381)
38     ductal carcinoma in [situ.mp](http://situ.mp/). (4453)
39     [dcis.mp](http://dcis.mp/). (3227)
40     37 or 38 or 39 (7090)
41     36 and 40 (7)
42     Prostatic Neoplasms/ (80689)
43     prostate [cancer.mp](http://cancer.mp/). (70257)
44     42 or 43 (90868)
45     36 and 44 (491)
46     limit 45 to (english language and yr="2006 -Current") (347)
47     Kidney Neoplasms/ (33721)
48     renal [cancer.mp](http://cancer.mp/). (3046)
49     47 or 48 (34493)
50     36 and 49 (44)
51     Leukemia, Lymphocytic, Chronic, B-Cell/ (10405)
52     chronic lymphocytic [leukemia.mp](http://leukemia.mp/). (8706)
53     Lymphoma, Follicular/ (3717)
54     51 or 52 or 53 (15638)
55     36 and 54 (28)
