# Supplementary material for: Patient and provider experiences with active surveillance: A scoping review
Source: PLoS One. 2018 Feb 5;13(2):e0192097. doi: 10.1371/journal.pone.0192097 (PMC5798833; doi:10.1371/journal.pone.0192097)
Supplement: S1 Table — (DOCX) [file pone.0192097.s003.docx]

S1 Table. Data extracted from included studies

**DCIS** (n=2)

| Study  Focus | Research Design | Objective | Participants | Results |
| --- | --- | --- | --- | --- |
| McCaffery [24]  2015  Australia  Label for DCIS | Comparative cohort | Investigated the effect of describing DCIS as ‘abnormal cells’ vs. ‘pre-invasive breast cancer cells’ on women’s concern and treatment preferences | 269 Australian women  40.5% were aged 50 to 69 and 30.1% completed high school | - Women in both arms indicated high concern, but still indicated strong initial preferences for watchful waiting (64%). - More women in arm A ('abnormal cells' first term) indicated they would feel more concerned if given the alternative term ('pre-invasive breast cancer cells') compared to women in arm B who received the terms in the opposite order (67% arm A vs 52% arm B would feel more concerned, p=0.001). - More women in arm A also changed their preference towards treatment when the terminology was switched from 'abnormal cells' to 'pre-invasive breast cancer cells' vs arm B. - There were no statistically significant differences in treatment preferences between arm A (‘abnormal cells’) and arm B (‘pre-invasive breast cancer cells’) for women (33% and 41% of women, respectively, favouring treatment, p=0.23). - In arm A 18% of women changed their preference to treatment while only 6% changed to watchful waiting (p=0.008). In contrast, there were no significant changes in treatment preference in arm B when the terminology was switched (9% vs 8% changed their stated preference, p>0.99). |
| Nickel [25]  2015  Australia  Label for DCIS | Qualitative study  (interviews) | Investigated how different proposed terminologies for DCIS affected women’s perceived concern and management preferences | 26 Australian women ranged between 25 and 80 years with a mean age of 56 years.  Over 40% left school before or at the end of high school | - Women’s reaction to the cancer vs non-cancer terminology   - Diagnosis of DCIS elicited high concern regardless of the terminology used to describe it. Women generally exhibited stronger negative reactions when a cancer term was used to describe DCIS compared to a non-cancer term, and most preferred the diagnosis be given as a description of abnormal cells. - Their responses to other suggested terminologies   - The word “carcinoma” prompted a strong negative response. Most women indicated they would feel overwhelmed by unfamiliar terminology but when presented with the “abnormal cells” description, overall women appeared more comfortable about that term as it provided more information in plain language. - Their attitudes towards watchful waiting as a management option   - Overall women expressed interest in watchful waiting for DCIS but displayed preferences for very frequent monitoring with this management approach. |

**Chronic lymphocytic leukemia** (n=4)

| Study  Focus | Research Design | Objective | Participants | Results |
| --- | --- | --- | --- | --- |
| Holtzer [26]  2015  The Netherlands  WW | Questionnaire | To describe the health-related quality of life of patients with chronic lymphocytic leukaemia (CLL) including those undergoing watchful waiting | 144 patients were invited from 19 hospitals in the Netherlands of which 59 patients were in the watchful waiting group | - 85 patients received active treatment (59%). - Both patients in the watch and wait phase and those during treatment with chlorambucil scored lower on the visual analogue scale than the general population corrected for age and gender distribution. - Scores on the European Quality of Life 5 dimensions questionnaire (EQ-5D5) were significantly different between the categories of gender, age at diagnosis, World Health Organization performance status, and the presence/absence of comorbidities for patients during the watch and wait phase. - Patients in the watchful waiting phase suffered most from worries about their future health (55% of the questionnaires), night sweats (44%), and having not enough energy (40%). - The health-related quality of life was significantly worse during treatment with chlorambucil than during the watchful waiting phase for the following outcomes: utility, visual analogue scale, emotional functioning, social functioning, fatigue, dyspnoea, losing weight, changes in temperature, feeling apathetic, lack of energy, respiratory infections, and risk of infections |
| Van den Broek [27]  2015  The Netherlands  AS | Questionnaire | To assess health-related quality of life among patients undergoing active surveillance or treatment | Of 175 patients diagnosed with chronic lymphocytic leukaemia/small lymphocytic leukaemia between 2004 to 2011 identified from the Netherland Cancer Registry, 138 (78%) returned the health-related quality of life questionnaire | - Compared to patients under AS, patients having received any type of chemo- and/or immunotherapy reported worse scores on physical and role functioning and had more financial problems. - The prevalence of fatigue among the chlorambucil group (81%) was almost twice as high compared to that of the AS group (42%) (p<0.01). - Patients related with chlorambucil were also more worried about the future, their health, the cancer coming back, and the occurrence of new symptoms than patients in the AS group. - No difference was observed for anxiety and depressive symptoms between any of the treatment groups. |
| Evans [28]  2012  UK  WW | Qualitative  (interviews) | Explored how people understood and interpreted the meanings of their chronic lymphocytic leukaemia diagnosis and of watchful waiting | 12 people with chronic lymphocytic leukaemia managed by watchful waiting from sample of 39 adults with leukaemia in the United Kingdom  11/12 people identified as being retired | - Patients with chronic lymphocytic leukaemia recalled being given little information about the condition and wanted to know more about how it might affect them in the future. - The invisibility of chronic lymphocytic leukaemia meant that some chose not to disclose the diagnosis to others. - Check-ups sometimes felt cursory, causing dissatisfaction. - As symptoms increased, lifestyle adaptations became essential, well before treatment was warranted. - Patients with chronic lymphocytic leukaemia on watchful waiting experience levels of depression, anxiety and quality of life similar to those in active treatment. |
| Levin [29]  2007  USA  WW | Questionnaire | Examined anxiety, depression and quality of life in a chronic lymphocytic leukaemia cohort | 105 patients above the age of 18 and were fluent in English were recruited from a chronic lymphocytic leukaemia research database (previously consented to give a blood sample for a tissue bank) classified into 2 groups: watch and wait or active treatment | - There was no statistical difference between depression, anxiety and physical/mental quality of life in watchful waiting vs active treatment. - Patients younger than or equal to 60 reported more depression (p=0.014) and worse emotional (p=0.0001) and social quality of life (p=0.002). They also had more “watch and wait” anxiety (p=0.052). - Social and emotional quality of life were similar in both newly diagnosed patients and those diagnosed more than 6 years ago, although physical quality of life worsens with time (p=0.05). |

WW watchful waiting; AS active surveillance

**Renal Cell Cancer** (n=6)

| Study | Research Design | Objective | Participants | Results |
| --- | --- | --- | --- | --- |
| Maurice [30]  2015  USA  observation | Single cohort | To determine the rate of observation utilized over time and to identify factors influencing its use | Of 109,410 analyzed patients, 7047 (6.4%) underwent observation  Patients were diagnosed between 2003 to 2010 using the National Cancer Database  27% of the cohort were 55 to 64 years of age | - Patient disease factors were the strongest predictors of observation. Specifically, the odds of biopsy were 1.8-11 times higher for elderly or comorbid patients and 1.6-8.4 times higher for small (clinical T1a), biopsied, or bilateral tumors (p <0.01 for all). - Racial and socioeconomic factors also significantly predicted observation usage. In particular, observation rates were higher among poor, African American, and uninsured or socially insured patients, with these groups having 1.2 to 3.5 times higher odds of observation (OR, 3.45; CI, 2.92 to 4.09 and OR, 1.76; CI 1.62 to 1.91; p<0.01). - Patients receiving care at community, low-volume, or nearby hospitals were also significantly more likely to undergo observation (p <0.01). |
| Parker [31]  2013  USA  WW | Single cohort | To examine the influence of uncertainty on general and cancer-specific quality of life and distress in patients undergoing watchful waiting for a renal mass | Between 2006 and 2010, 264 patients from a large comprehensive cancer center were recruited; the first 100 participated in this study  At the time of enrollment, the mean age was 72.5 years (standard deviation [SD]: 9.9; range: 47.8 to 91.1)  55% of the patients were male, and 84% were Caucasian. | - Growth rate was an average of 0.17cm/yr. - Greater illness uncertainty was associated with poorer general quality of life scores in the physical domain (p=0.008); worse cancer-related quality of life in physical (p=0.001), psychosocial (p <0.001), and medical (p = 0.034) domains; and higher distress (p <0.001). - Illness uncertainty predicted general quality of life, cancer-specific quality of life, and distress. |
| Smaldone [32]  2013  USA  AS | Comparative cohort | To determine the associations between the pre-treatment characteristics and treatment selection in patients presenting with clinical stage I renal masses | Using institutional data (kidney tumor database), patients with clinical stage I (< or = 7cm) renal tumors that were managed with AS (25.7%), tumor ablation (6.1%), partial nephrectomy (38.8%), or radical nephrectomy (29.4%) from 2005 to 2011 were identified.  266 patients on AS out of a total of 969 patients (mean age 61.9). | - The use of AS did not significantly change from 2005 to 2010 (12% vs 20%, p=0.09) vs. the use of partial nephrectomy significantly increased (37% vs 68%, p <0.0001), the use of radical nephrectomy (38% vs 7%, p=0.02) and tumor ablation (12% vs 5%, p<0.001) significantly decreased over time. - Traditionally captured covariates, including older age (partial nephrectomy, OR 0.96, 95% CI 0.94 to 0.99) and decreasing tumor size (partial nephrectomy, OR 0.2, 95% CI 0.1 to 0.4; tumor ablation, OR 0.01, 95% CI 0.0 to 0.1; AS, OR 0.2, 95% CI 0.1 to 0.3) were associated with alternative treatment types compared with radical nephrectomy. - The characteristics associated with treatment type that are not included in traditional registry or administrative data included the presence of a solitary kidney (partial nephrectomy, OR 11.9, 95% CI 2.9 to 48.9; tumor ablation, OR 15.5, 95% CI 2.5 to 98.1; AS, OR 7.1, 95% CI 1.3 to 39.3) and high complexity nephrectomy score (partial nephrectomy, OR 0.1, 95% CI 0.1 to 0.3; tumor ablation, OR 0.1, 95% CI 0.0 to 0.6; AS, OR 0.1, 95% CI 0.03 to 0.3). - AS was the primary management strategy use in 22% of patients with stage I tumors at their institution, a trend that remained consistent throughout the study period. |
| Sun [33]  2012  Canada  AS | Comparative cohort | Examined the rates of partial and radical nephrectomy and AS within a contemporary population based cohort | 26,468 patients diagnosed with T1aNoMo renal cell carcinoma were identified from the surveillance, epidemiology and end results database.  Patients were aged between 51 to 78 (median, 60) years.  White race (84%) and male gender predominated (60%). | - 8,966 (34%) underwent partial nephrectomy. 14,705 (56%) underwent radical nephrectomy. 2,797 (11%) underwent AS. - The rate of partial nephrectomy increased (4.7% in 1988 to 40.4% in 2008, p <0.001), whereas the rate of radical nephrectomy decreased over time (92.9% in 1988 to 41.4% in 2008, p <0.001). The rate of AS increased over time (2.4% in 1988 to 18.2% in 2008, p <0.001). - The determinants for partial nephrectomy consisted of more contemporary year of diagnosis, younger patient age, male gender, Caucasian race, married status, and decreasing tumor size (all p < or = 0.003) vs. the determinants of AS consisted of more contemporary year of diagnosis, more advance age, male gender, decreasing tumor size, and unmarried marital status (all p < or = 0.001). |
| Jacobs [34]  2012  USA  AS | Single cohort | To better delineate which factors influence the decision to undergo AS of small renal masses | 204 consecutive patients with clinical stage T1 renal masses (≤ 7cm) referred to the institution from June 2009 to June 2010  73 (36%, mean age 63) underwent AS and 131 (64%, mean age 60) underwent treatment. | - Patients undergoing AS vs treatment differed with respect to distance from hospital >60 miles (p=0.04), Eastern Cooperative Oncology Group performance status (ECOG PS) of ≥ to 2 (p <0.1), tumor size (p <0.01), multifocality (p=0.03), endophytic nature of lesion (p=0.04), and whether the patient's surgeon generally used a robotic, laparoscopic, or open approach (p=0.01) - The combination of tumor size <3cm, Eastern Cooperative Oncology Group performance status of ≥ 2, and an endophytic lesion were most predictive of AS. - Patients treated by surgeons who performed primarily open surgical partial nephrectomy were more likely to enter AS (p=0.01). - Surgeons primarily using an open approach were more likely to choose AS than those using a robotic approach (OR 4.47, 95% CI 1.76 to 11.37). |
| Breau [35]  2011  USA  AS | Questionnaire | Surveyed American Urological Association members to determine factors that influence the treatment of patients with small renal masses | Of 14,749 American Urological Association members, 4,513 (31%) with registered e-mail addresses were invited to participate. Of those, 866 (19%) completed the survey and 759 were included in analyses.  234 (30.8%) of the urologists were aged 51 to 60 years. 556 (73.8%) were located in North America. 307 (40.5%) had their practice setting in an academic hospital. | - Respondents were more likely to choose AS (compared to any other treatment) in an older patient (OR 2.7; 95% CI 2.1, 3.6; p <0.0001) or in a patient with comorbidities including renal dysfunction (OR 10.0; 95% CI 8.0, 12.4; p <0.0001). - Urologists were less likely to choose AS of a 4 cm tumor compared to a 2 cm tumor (OR 0.18; 95% CI 0.15, 0.21; p <0.0001). - AS was chosen more often if the tumor was perihilar compared to mid kidney (OR 2.0; 95% CI 1.8, 2.3; p <0.001)or polar (OR 2.1; 95% CI 1.9, 2.5; p <0.0001) |

**Prostate Cancer** (n=65)

| Study | Research Design | Objective | Participants | Results |
| --- | --- | --- | --- | --- |
| Bayliss [36]  2016  Australia  AS | Qualitative  (interviews) | To understand why and how men choose AS | 4 patients who had been recruited through purposive sampling and were essentially health young men who had chosen AS were recruited. | - The surgeon or general practitioner’s recommendation as the most influential factor when making a decision - Being able to trust the doctor was important; although all patients denied researching different treatments, they all researched the surgeon both electronically and by word of mouth - When deciding on a treatment, patients discussed the need to consider potential side effects, and the avoidance of these was often a significant factor when deciding whether to pursue AS or other forms of treatment - All patients placed a high value on their sexual health, thus avoiding erectile dysfunction (ED) was an important factor - Participants were relieved when told that cancer was low-grade and viewed their cancer as a non-issue. |
| Parker [37]  2016  USA  AS | Single cohort | To examine the psychosocial adjustment and Quality of Life of men on AS and to examine the associations between illness uncertainty and quality of life, anxiety, and fear of progression | 180 men with low-risk prostate cancer who were enrolled during 2006-2012 in the AS protocol at the University of Texas MD Anderson Cancer Center were included in this study.  The mean age was 67.2 years with a range of 40 to 87 years. 155 (86.1%) of the men were white. | - At a 2.5 year follow-up, quality of life remained stable. - Sexual scores on the Expanded Prostate Cancer Index Composite (EPIC) scale significantly declined over time (P<0.05) - Illness uncertainty was a significant predictor of all EPIC summary scores, SF-12 physical component summary (PCS) scores, mental component summary (MCS) scores and fear of progression scores (all P<0.05), after controlling for demographic and clinicopathological factors. - Anxiety predicted all EPIC summary, MCS and fear of progression scores (all P<0.05) but not PCS scores (P=0.08). - Scores on PCS, MCS, EPIC summary scales (except sexual scale), and fear of progression did not change significantly over the study period (all P>0.10). |
| Hurwitz [38]  2016  USA  AS | Single cohort | Understanding how and why patients make their treatment decisions, as well as the effect of treatment choice on long-term outcomes, is critical to ensuring effective, patient-centered care | 925 patients with prostate cancer at the Walter Reed National Military Medical Center  Mean age was 61 years old  59% were white  86% were married  52% had a masters/doctoral degree | - Surgery (54%), external radiation (20%), and AS (12%) were the most common primary treatments for patients with low- and intermediate-risk prostate cancer. - The use of AS increased significantly since study onset in 2006 (6%) to 2014 (31%). - After multivariable adjustment, age (P<0.0001), race (P<0.0001), comorbidity status (P=0.02), and calendar year (P<0.0001) remained significant, independent predictors of treatment choice. - Most patients (68%) preferred to play an active role in the decision-making process and cited doctors at the clinic as the most helpful source of treatment-related information (87%). - Almost all patients (98%) reported satisfaction with their treatment decision. |
| Loeb [39]  2016  USA  AS | Qualitative (interviews) | To explore and identify factors that influence physicians’ decisions while monitoring patients with prostate cancer on AS | E-mail invitations were sent to 48 physicians, 24 interviews were conducted  The median age was 43 years old with a range of 33 to 70  91.7% were men and 62.5% were white  83.3% were urologists and 75% practiced at an academic setting | - Physician comfort with AS: All participants stated that among the biggest advances in AS was the availability of long-term outcomes data, which made them more comfortable recommending AS. All physicians agreed how AS is now a standard management approach and not experimental - Protocol selection: For patient selection, there was 100% agreement on grade, and nearly all participants also used prostate specific antigen and clinical stage. There was less agreement on the use of age, imaging and other tests for patient selection or monitoring. - Beliefs about the utility and quality of testing: All physicians stated that they rely heavily on biopsy results and most felt prostate specific antigen was less important. - Years of experience and exposure to AS during training: Physicians’ feelings regarding AS varied depending on how long ago they trained. Physicians who completed training recently tended to follow specific protocols more strictly, and several reported being uncomfortable with the lack of national guidelines. - Concerns about inflicting 'harm': Nearly all participants mentioned the tension between ‘over-‘ or ‘under-‘ testing patients and the desire to reduce ‘harm’ whether through repeat biopsies vs the risk of ‘missing’ aggressive disease. - Patient characteristics: Most physicians consider patient characteristics such as age, comorbidities, and history of compliance when deciding who is an appropriate candidate for AS and when it should be discontinued. - Patient preferences: Several participants mentioned that the ‘type’ of patients choosing AS tend to be more comfortable with ‘uncertainty’ and regular follow-up testing. - Financial incentives: Physicians did not believe that financial incentive directly affected their individual practice or their decisions while monitoring men on AS, but many acknowledged that it may influence a physician’s decision to recommend AS. Some physicians felt that financial incentives were a reason why the uptake of AS in the US has been slower than in other countries. |
| Pham [40]  2016  USA  AS | Comparative cohort | Evaluated health related quality of life outcomes in men on AS compared to men followed after negative prostate needle biopsy | Of the 1,204 men who met the initial eligibility criteria 420 (35%) has a negative prostate needle biopsy (noncancer comparison group).  Among the 411(52%) men diagnosed with low risk prostate cancer 89 were on AS.  Mean age at baseline was 61 and 64 years in the noncancer and AS groups, respectively (p=0.0004) | - For most health related quality of life subscales there were no significant differences between the groups in adjusted health related quality of life score trends over time. - Patients on AS underwent more prostate needle biopsy’s following baseline (p<0.0001) and were more likely to be Caucasian (p=0.01). - Twelve subjects on AS received secondary treatment (radical prostatectomy in 6, radiation therapy in 5 and hormonal therapy in 1) after a median of 25 months. - Survey completion rates remained modest for the noncancer and AS groups at 36 months (61% and 67%, respectively) - After adjustment for baseline health related quality of life and other potential confounders, only mean bowel functional at 12 months remained statistically significantly different between the groups (91 vs 95 for noncancer and AS groups, respectively, p=0.01) - Patients on AS experienced declines in urinary function (p=0.002), bowel function (p=0.01) and bodily pain (p=0.03). - Only urinary function (p=0.047) and mental health (p=0.048) time trends were statistically significantly different between the groups. |
| Tan [41]  2016  USA  AS | Single cohort | This study explored prospectively the relationship of intolerance of uncertainty with anxiety along with other clinical factors among men treated with AS for prostate cancer | 119 men with low risk prostate cancer participating in AS  Mean age 63.2 years  Majority of the participants were white | - Of the men 18 (15.1%) and 17 (14.3%) reported clinically significant anxiety on the generalized and prostate cancer specific scales, respectively. - Men with moderate/severe urinary symptoms and higher Intolerance of Uncertainty Scale (IUS) scores reported more generalized and prostate cancer specific anxiety than men with mild urinary symptoms and lower IUS scores, respectively (p≤0.008). - Men with depressive symptoms (p=0.024) or a family history of prostate cancer (p=0.006) experienced greater generalized anxiety. - On multivariable analysis IUS score was significantly associated with generalized and prostate cancer specific anxiety (OR 1.22, 95% CI 1.09 to 1.38 and OR 1.29, 95% CI 1.13 to 1.49, respectively) while moderate/severe urinary symptoms were associated with prostate cancer specific anxiety (OR 6.89, 95% CI 1.33 to 35.68). |
| Taylor [42]  2016  USA  AS | Qualitative (interviews) | To comprehensively assess factors associated with treatment decision-making for low-risk prostate cancer | 1140 men with newly diagnosed low-risk prostate cancer  Men were 61.5 years older, 24 days (median) after diagnosis, and 81.1% white. | - Treatment preferences were: 39.3% AS, 30.9% active treatment, and 29.7% no preference. - Multinomial logistic regression revealed that men preferring AS (vs. active treatment) were older (OR, 1.64; CI, 1.07 to 2.51), more educated (OR, 2.05; CI, 1.12 to 3.74), had greater prostate cancer knowledge (OR, 1.77; CI, 1.43 to 2.18) and greater awareness of having low-risk cancer (OR, 3.97; CI, 1.96 to 8.06), but also were less certain about their treatment preference (OR, 0.57; CI, 0.41 to 0.8), had greater prostate cancer anxiety (OR, 1.22; CI, 1.003 to 1.48), and preferred a shared treatment decision (OR, 2.34; CI, 1.37 to 3.99). - Similarly, men preferring no preference (vs. active treatment) were less certain about treatment preference, preferred a shared decision, and had greater knowledge. |
| Chu [43]  2015  USA  AS | Comparative cohort | To evaluate the impact of the urologist’s experience in selecting AS vs immediate treatment | 4754 men were diagnosed with prostate cancer.  713 of the men were included in the study.  87 urologists were included in the study.  Mean age of the patients was 61.8 years. 50.5% were white. 76.7% were married/partnered.  256 patients reported their urologist being between the ages of 50 to 70 | - 433 (60.7%) men chose AS and 280 (39.3%) men chose immediate treatment. - Patients who saw urologists who had performed ≥50 robotic surgeries were less likely to choose AS (OR 0.40, 95% CI 0.25 to 0.66). - Patients who saw urologists with a fellowship in oncology and/or robotics were more than twice as likely to choose AS (OR 2.27, 95% CI 1.38 to 3.75). - Urologist’s age or years in practice made no difference |
| Wade [44]  2015  UK  AS | Comparative cohort | To develop a nurse-led, urologist-supported model of care for men managed by AS/AM (active monitoring) for localised prostate cancer and provide a formative evaluation of its acceptability to patients, clinicians and nurses. | 22 men receiving active monitoring, 11 urologists, and 23 research nurses delivering ProtecT trial care completed a questionnaire about its acceptability.  20 men managed in urology clinics elsewhere in the UK, 12 urologists, and 3 specialist nurses were interviewed about models of AS/active monitoring care. | - Nurse-led care was commended by ProtecT trial participants, who valued the flexibility, accessibility and continuity of the service and felt confident about the quality of care. - ProtecT consultant urologists and nurses also rated it highly, identifying continuity of care and resource savings as key attributes. - Clinicians and patients outside the ProtecT trial believed that nurse-led care could relieve pressure on urology clinics without compromising patient care. - Three key themes emerged in support of nurse-led care:  1. Efficient use of resources 2. Quality of care 3. Convenience of care  - A majority of men receiving consultant-led AS (N=12) and a majority of clinicians or nurses involved in delivering AS outside the ProtecT trial (N=9) supported alternative to the consultant-led model, citing the reasons above and also suggesting that:  1. Nurse-led care was a natural extension of existing nurse practice |
| Kayser [45]  2015  Denmark  AS | Single cohort | To explore whether the scores of and verbal responses to a Health Literacy Questionnaire can be used to identify individuals in need of information and support and to reveal differences in perception and understanding in health related situations within couples | 16 participants: 8 men on AS and their spouses  Age range of the men were 55 to 70 years, age range of their spouses were 55 to 68  The majority in both groups were employed | - Men tended to score higher than their spouses. - There was no consistent relation between scores and reported experiences and reflections - In two of the three couples with the largest within couple differences in Health Literacy Questionnaire scores, responses revealed discrepancies in how the men and their spouses perceived their situation - 3 themes emerged which related to 6 of the HLQ domains:  1. Involvement of spouses and other people around the men: most men expressed a wish to be discrete about their health condition 2. Support from and interaction with healthcare professionals 3. Use of the Internet for information retrieval: most of the men often used the Internet for prostate cancer information retrieval  - The mean scores of all participants for the first four domains were 3.3 and 4.0 to 4.2 for domains six to nine. - Domain V: ‘Appraisal of health information’ recorded the lowest score of 3.0 - The largest difference was observed in domains I: ‘Feeling understood and supported by healthcare providers’ (0.5 unit difference) and III: ‘Actively managing my health’ (0.4 unit difference). - For the remaining domains, men tended to score slightly higher or the same (0.0 to 0.2 unit difference), except for domain V: ‘Appraisal of health information,’ where men scored 0.2 units lower than women. |
| Watts [46]  2015  UK  AS | Single cohort | To quantitatively determine the prevalence of anxiety and depression in men on AS | 313 men on AS with a histological diagnosis of prostate cancer were identified from seven United Kingdom urology departments  The mean age of respondents was 70 years with a range of 51 to 86 years.  The majority (76%) were married or in civil partnerships and 94% were of white British ethnicity. | - The prevalence of clinical anxiety and depression as determined via the Hospital Anxiety and Depression Scale (HADS ≥8) was 23% (n=73) and 12.5% (n=39), respectively. - Published data from men in the general population of similar age has shown prevalence rates of 8% and 6% respectively, indicating a twofold increase in anxiety among AS patients. - AS patients experience substantially greater levels of anxiety than patients with prostate cancer treated radically. - The only demographic predictor for anxiety or depression was divorce. |
| Yanez [47]  2015  USA  AS | Single cohort | Examined whether two types of perceived stress management skills, specifically the ability to relax and confidence in coping, moderated the relationship between prostate cancer concerns and psychological distress | 71 men on AS  Mean age was 65.4 years.  37 participants were non-Hispanic white, 22 were Hispanic, and 12 were African American/Black  42 were married/equivalent relationship | - Coping confidence moderated the relationship between prostate cancer concerns and intrusive thoughts (p<0.01). - At low levels of coping confidence, prostate cancer concerns was positively related to intrusive thoughts, p<0.001but not when coping confidence was high, p>0.05. - Coping confidence also moderated the relationships between prostate cancer treatment concerns (a subscale of prostate cancer concerns) and intrusive thoughts. - At low levels of coping confidence, prostate cancer treatment concerns was positively associated with intrusive thoughts, p<0.001, but not when coping confidence was high, p>0.05. |
| Volk [48]  2015  USA  AS | Questionnaire | To test the acceptability of normative messages about AS as a management option for men with clinically localized PC from the perspectives of men who have made a treatment decision | 331 respondents with completed questionnaires had an average age of 54.9 years, range 45 to 96 years  88% classified themselves as white, 82% were married, and 93% were college educated  Treatment history included surgery (37%), AS (24%), radiation (26%), and other (13%). | - For the believability dimension, ratings of messages largely ranged from 65.1% (message 4, representing trust in the AS protocol to catch any progression in time for curative treatment) to 90.1% (message 8, regret over not giving AS a second thought). - Message 9, a favorable quote about AS attributed to a surgeon, was a negative outlier on the believability dimension. - Ratings of message accuracy varied more widely, with 36.8% (message 4) to 85.6% (message 1, having time to think about options) of respondent judging the various messages to be accurate. - Men’s ratings of the utility of different messages ranged from 36% (message 9) to 82.9% (message 1). - The message “you don’t have to panic… you have time to think about your options” (message 1) was perceived as believable, accurate, and important for men to hear by more than 80% of respondents. - More than 75% of respondents rated as believable and accurate a message about having low-risk prostate cancer and “not wanting to rush into getting something done right away,” (message 3) and 66% rated it very important for men to hear. - Messages about trust in the AS protocol and “knowing in plenty of time” if treatment is needed (message 4) were rated as accurate by only about 35% of participants. - Message 9, in which a surgeon describes men who chose AS as “jubilant” and “delighted” with their lack of side effects, received consistently low ratings in ever dimension: believability, 13.5%; accuracy, 36.9%; utility, 36%. - Perceptions of the effect of each message on men’s likelihood of choosing AS ranged from 60.4% (message 1) to 77.3% (message 6, prolonging the decision until treatment options have improved). - The outlier was message 8 (regret over not giving AS a second thought), which was viewed as favoring an AS decision by 45.1% of respondents. |
| Jeldres [49]  2015  Canada  AS | Comparative cohort | Examining health-related Quality of Life between AS and radical prostatectomy | Out of 389 men with low-risk prostate cancer 228 (58.6%) underwent radical prostatectomy and  77 (19.8%) underwent AS  Mean age in the AS group was 65 years with a range of 45 to 75 years and 61 (70%) participants were Caucasian  Mean age in the radical prostatectomy group was 58 years with a range of 40 to 74 years and 163 (71%) were Caucasian | - At baseline, the AS cohort reported lower sexual function and bother scores (p=0.002 and p=0.03, respectively). - Adjusted mean sexual function and bother scores were lower for radical prostatectomy patients at all follow-up time points. In the radical prostatectomy cohort, sexual function and bother scores improved after 6 months, stabilized by 2 years, but remained significantly lower than those for AS patients at 3 years (p=0.01 and p=0.049, respectively). - Compared with the AS cohort, men in the radical prostatectomy cohort reported significantly poorer urinary function at all follow-up time points. Urinary function scores recovered between 3 months and 12 months in the radical prostatectomy cohort but remained lower than in the AS cohort at 3 years (p=0.046). - Differences in mental health between groups were below the threshold for clinical significance at one year |
| Venderbos [50]  2015  The Netherlands  AS | Single cohort | To analyse how anxiety and distress develop in men with untreated prostate cancer and whether high anxious men quit AS | 129 patients managed with AS  Mean age at diagnosis was 64.6 years with a range of 60.2 to 70.4 years  86 (675) had low (primary, secondary) education, and 119 (92%) were married/living together. | - CES-D, total MAX-PC and DCS scores did not change significantly (p>0.05) when comparing t=18 with t=9 and t=0 scores, but generic anxiety (STAI-6; p=0.033) and fear of disease progression (sub-score of the MAX-PC; p=0.007) decreased significantly. - These differences, however, were clinically modest (0.089 SD and 0.281 SD). - Response rates for patients still on AS at t= 0, t= 9, t= 18 assessments were 86%, 90%, and 96% respectively. - Generic anxiety and fear of disease progression decreased significantly. - Overall, 6 of 129 men (5%) discontinued AS because of anxiety and distress |
| Ravi [51]  2014  UK  WW | Single cohort | To examine the burden of mental health issues, namely anxiety, depressive disorders, and suicide, in a population based cohort of older men with localized prostate cancer and to evaluate associations with primary treatment modality | 50,856 men, who were 65 years of age or older with clinically localized prostate cancer diagnosed between 1992 to 2005 and without a diagnosis of mental illness at baseline.  Mean age was 72 years with a range of 69 to 75 years  44,098 (86.7%) were white and 38713 (76.1%) were married.  13,186 (25.9%) underwent watchful waiting, 12,191 (24%) radical prostatectomy, and 25,479 (50.1%) radiotherapy | - 10389 (20.4%) patients developed mental health issues. It is notable that patients treated with "definitive" therapy, radical prostatectomy (2471 (23.8%) or radiotherapy (5137 (49.4%) had lower risk of developing mental health issues compared to watchful waiting (2781, 26.8%). - This may indicate greater psychological distress among patients undergoing watchful waiting, as this cohort has been shown to have high levels of uncertainty on treatment choice and lower satisfaction on choosing this treatment path. Men undergoing watchful waiting were more likely to be older and single, married, or divorced. - The watchful waiting cohort also had a higher proportion of men who were of black or other race, which was an independent predictor of a lower risk of mental health issues. - Independent risk factors for MHI included age ≥75 years (hazard ratio [HR] = 1.29); higher comorbidity (Charlson comorbidity index ≥3, HR = 1.63); rural hospital location (HR =1.14); being single, divorced, or widowed (HR = 1.12); later year of diagnosis (HR = 1.05); and urinary incontinence (HR = 1.47). - Black race (HR = 0.79), very high-income status (HR = 0.87), and definitive treatment (RP, HR = 0.79; RT, HR = 0.85, all p<0.001) predicted a lower risk of mental health issues. - The rates of mental health issues at 10 years were 29.7%, 29%, and 22.6% in men undergoing watchful waiting, radiotherapy, and radical prostatectomy, respectively. |
| O’Callaghan [52]  2014  Australia  AS | Qualitative  (interviews) | To examine men with localised prostate cancer and their partners’ experiences of choosing between AS and radical treatments and their experiences of AS when selected | 21 men and 14 partners  11 of the men were between the ages of 61 to 70 years and 7 of the partners were between the same age range as well  16 men had post high school qualifications and 10 of the partners had the same qualification | - Treatment decisions reflected varied reactions to prostate cancer information, regularly described as contradictory, confusing, and stressful. - Men and partners commonly misunderstood AS but could describe monitoring procedures. - Partners often held the perception that they were also on AS. - Men and partners usually coped with AS but were sometimes encumbered by treatment decision-making memories, painful biopsies, ongoing conflicting information and unanswered medical questions. - Radical treatment was selected when cancer progression was feared or medically indicated - Some preferred doctors to select treatments |
| Anderson [53]  2014  Australia  AS | Single cohort | To describe a range of anxieties in men on AS for prostate cancer and determine which of these anxieties predicted health related quality of life | 260 men with prostate cancer on AS were invited to complete the questionnaires  86 men returned data (33%)  Mean age of men was 65.7 years with a range of 51 to 75 years.  64 (74.4%) men were born in Australia. 57 (66.3%) were living with their spouse/partner without children. 54 (62.8%) worked for themselves and 38 (44.2%) worked full time. | - Most men in the present sample had normal levels of general state (86%) and trait (77%) anxiety. - Most men exhibited low levels of illness-specific prostate cancer anxiety (87%), Fear of recurrence [FoR] (92%) and prostate specific antigen (99%) anxiety. - Participants reported low levels of anxiety and high HRQoL. - Trait anxiety and FoR were significant predictors of both PC-related and total HRQoL - 23 men ceased AS during follow-up, of these, 4 men ceased AS through choice rather than as a result of clinical progression - These men has a mean (range) age of 66.5 (64 to 69) years, mean prostate specific antigen level of 6.43ng/mL, and a median (range) time on AS of 6 (5 to 62) months. - Although they reported lower state anxiety, patients who chose to cease AS had higher levels of prostate cancer-specific anxiety and FoR. |
| Kim [54]  2014  USA  AS | Questionnaire | To assess opinions of radiation oncologists and urologists about their perceptions of AS and treatment recommendations for low-risk prostate cancer | 717/1366 (52.5%) radiation oncologists and urologists in the United States completed the survey  Of the 361 radiation oncologists, 46.8% were between the ages of 40 to 54, 85.3% were white and 81.4% were male.  Of the 356 urologists, 48% were between the ages of 40 to 54, 84.6% were white and 94.4% were male. | - Although, most physicians reported that AS is effective (71.9%) and underused in the United States (80%), (71%) stated that their patients were not interested in AS. - For low-risk prostate cancer, more physicians recommended radical prostatectomy (44.9%) or brachytherapy (35.4%); fewer endorsed AS (22.1%). - Urologists were more likely to recommend surgery [OR: 4.19; p<0.001] and AS (OR: 2.55; p<0.001), but less likely to recommend brachytherapy (OR: 0.13; P<0.001) and external beam radiation therapy (OR: 0.11; P<0.001) compared with radiation oncologists. - Physicians practicing in academic medical centers had higher adjusted ORs for recommending AS compared with those practicing in the community setting (OR: 2.35; 95% CI, 1.42 to 3.89; p<0.001). |
| Wilcox [55]  2014  Australia  AS | Single cohort | To assess anxiety, health related quality of life and understanding of AS in a cohort of patients enrolled in AS of prostate cancer in an Australian setting | 47/61 (77%) men on AS responded  The group of eligible patients (61) had a mean age of 62 years. | - The respondents (47) had mean (95% CI) scores on International Prostate Symptom Severity Score (IPSS) of 9.4 (7.5 to 11.3) and International Index of Erectile Function (IIEF-5) of 18.3 (16.1 to 20.4). - The mean (95% CI) overall Memorial Anxiety Scale for Prostate Cancer (MAX-PC) among the respondents was 15.5 (13.4 to 17.6) with subscale results of 7.4/33 for general anxiety (95% CI 5.5 to 9.3), 0.8/9 for PSA specific anxiety (95% CI 0.3 to 1.3) and 7.3/12 for fear of recurrence (95% CI 6.5 to 8.2) - There was no significant difference in patients’ IIEF-5 and IPSS scores at commencement of AS compared with the survey results. - Patients on AS MAX-PC scores were consistent with other published cohorts and did not suggest high rates of clinically significant anxiety amongst this cohort. - Most (89%) of the patients’ responses to the UAS indicated a correct understanding of AS. - The patients on AS maintained their health related quality of life with low levels of anxiety |
| Orom [56]  2014  USA  AS | Single cohort | To test the role of the quality of the physician-patient relationship in the choice to be followed by AS, rather than receive definitive therapy | 120 patients with prostate cancer on AS  107 (89.9%) participants were white and 87 (73.7%) were married or has partner  65 (54.6%) had ≥ college education, 42 (43.4%) had income ≥ $75,000, and 85 (70.8%) were retired or not employed. | - After controlling for the type of treatment recommended, Gleason score, and education, 3 predictors were associated with greater influence of physician’s treatment recommendation:  1. Trust in physician 2. Perceived closeness with the physician 3. The degrees to which the physician shared control over treatment decision making  - Receiving a recommendation for AS vs. definitive therapy was also associated with higher perceived trust (3.94/5), closeness (3.69/7), shared control over treatment decision making (64.6/100), and greater influence of physician’s treatment recommendation - Poor treatment by at least 1 prostate cancer physician was reported by 42.5% (51/120) of the sample - 113 (94.2%) has consulted more than 1 physician (urologist or radiation oncologist) about their prostate cancer - 87 (72.5%) received at least 1 recommendation for AS |
| Volk [57]  2014  USA  AS | Qualitative  (interviews) | To describe patients’ conceptualizations of AS and reasons for their treatment decisions | 15 men who chose AS  15 men who underwent radiation or surgery  Men in the AS group were on average 62.6 years old (range, 49 to 72 years), and men in the treatment group were on average 58.6 years old (range, 45 to 70 years).  Most men had at least some college education, all but two classified themselves as white, and three quarters were married. | - Men who chose AS described it as an organized process with a rigorous and reassuring protocol of periodic treating, with potential for subsequent and timely decision-making about treatment. - AS was seen as prolonging their current good health and function with treatment still possible later. - Rationales for choosing AS included trusting their physician’s monitoring, ‘buying time’ without experiencing adverse effects of treatment, waiting for better treatments, and seeing their cancer as very low risk. - Men recognize the need to justify their choice to others because it seemed contrary to the impulse to immediately treat cancer. - Descriptions of AS by men who chose surgery or radiation were less specific about the testing regimen. - Getting rid of the cancer and having a cure were paramount for them. |
| Acar [58]  2014  The Netherlands  AS | Comparative cohort | To investigate quality of life after different treatment modalities for lower-risk including brachytherapy, robot-assisted laparoscopic prostatectomy, and AS | 144 men with low-grade localized prostate cancer  65 (45.1%) with robot-assisted laparoscopic prostatectomy, 29 (20.2%) with brachytherapy, 50 (34.7%) with AS  The mean age of the total group was 61.9 years with a range of 44 to 85 years. | - In comparison with baseline scores, the brachytherapy group showed a significant decrease of quality of life domain scores of voiding complaints (p=0.010), use of incontinence aids (p=0.011), sexual functioning domain (p=0.011), and erectile function (p≤0.001). - In the robot-assisted laparoscopic prostatectomy group, sexual function (p≤0.001), incontinence (p≤0.001), and erectile function ere significantly affected. - A decrease in sexual function was observed in 71% of men after robot-assisted laparoscopic prostatectomy and 59% after brachytherapy. - In 30% of men under AS, a decrease of erectile function score during follow-up was reported. - Overall, no significant decrease in general quality of life was observed neither for men under AS nor for men treated by brachytherapy or robot-assisted laparoscopic prostatectomy. - Clinical factors such as age, prostate size, prostate specific antigen level, and nerve preservation during robot-assisted laparoscopic prostatectomy were nonpredictive of overall quality of life after treatment for the individual patient (p=0.676). |
| Berger [59]  2014  USA  AS | Qualitative (interviews) | To generate hypotheses about the factors that influence patients’ decisions to leave an AS program | Of 1159 men, 103 self-elected to leave AS  14/103 men who self-elected to leave AS were interviewed  69 (67%) of the men who self-elected to leave AS were between the ages of 60 to <70 years old, 97 (94%) were white, 38 (37%) had a graduate degree, and 69 (66%) were married or partnered | - Uncertainty involved in AS participation, existence of personal criteria - distinct from providers' clinical criteria - and fear of cancer were important factors in decisions to leave. - Men older than 70 years at diagnosis were significantly less likely to self-elect to leave AS than those aged between 40 to 60 years (relative risk ratio [RRR] 0.16, 95% CI 0.06 to 0.42). - The sentiment that “enough is enough,” i.e., that the uncertainties of biopsy results, together with worry generated by the presence of cancer, reached a point that leaving the program was preferable, was shared by a number of patients. - A total of six patients who self-elected to leave AS described a fear of cancer, which trumped clinical indications for surveillance of low-risk prostate cancer. |
| Womble [60]  2014  USA  AS | Single cohort | To describe contemporary utilization of AS among a population-based sample of men with low-risk prostate cancer | 682 men with low-risk prostate cancer  The mean age was 63 years with a range of 58 to 68 years old. 466 (68.3%) were between the ages of 55 to 69 years.  304 (49%) of men underwent initial AS | - Use of initial surveillance varied widely across practices (27 to 80%; p=0.005), even after accounting for differences in patient characteristics. - Among men undergoing initial surveillance with at least 12 months of follow-up, prostate specific antigen testing was common (85%), whereas repeat biopsy was performed in only one-third of patients. There was excellent agreement between treatment assignments in the MUSIC registry and claims data. - Older age, Charlson Comorbidity Index score ≥2, and fewer positive biopsy cores were associated with more frequent use of AS (all p values <0.05); specifically, 54% of patients ≥70 yr, 61% of patients with a Charlson score ≥2, and 57% of patients with only one or two positive cores received initial surveillance. |
| De-Bekker-Grob [61]  2013  The Netherlands  AS | Questionnaire | Investigated patients’ and urologists’ preferences for treatment alternatives for early prostate cancer | 110/150 (73%) patients and 50/150 (33%) urologists  Mean age of the patients was 72.9 years. 82% had a partner and 39% had intermediate education. 91% reported no anxious/depressed feelings  Mean age of the urologists was 49.2 years. 82% were male. | - Risk of urinary incontinence was an important determinant of both patients’ and urologists’ stated preferences for prostate cancer treatment (p<0.05). - Treatment modality also influenced patients’ stated preferences (p<0.05), whereas the risk of erectile dysfunction due to radiotherapy was mainly important to urologists (p<0.05). - Both patients and urologists preferred AS to radical treatment, with the exception of patients with anxious/depressed feelings who preferred radical treatment (59%) (radio therapy, p =0.05 or surgery, p=<0.01) to AS (41%) (p<0.01). - In contrast, patients who did not have anxious/depressed feelings had a lower probability to opt for radical treatment than AS (24% and 76%, respectively), which was quite similar to the choice probabilities of urologists (23% for radical treatment vs 77% for AS). |
| Punnen [62]  2013  USA  AS | Comparative cohort | To evaluate the prevalence of depression, anxiety and distress among AS and radical prostatectomy patients and evaluate the impact of these symptoms at baseline on urinary and sexual quality of life at follow-up | Of 874 men, 679 (77%) completed a baseline survey and were included in this study.  557 (82%) patients were managed with radical prostatectomy and 122 (18%) with AS  Mean age of radical prostatectomy group at diagnosis was 60 years, 508 (91%) were Caucasian, and 486 (87%) were married or partnered  Mean age of AS group at diagnosis was 60.5 years, 114 (93%) were Caucasian, and 101 (83%) were married or partnered. | - Baseline prevalence of moderate or higher levels of depression or anxiety were low (<5%), while levels of mild depression or anxiety ranged from 3 to 16% over time. - Baseline levels of elevated distress ranged from 8 to 20%. - Among men who provided data at baseline and follow-up, there were no significant differences between AS and radical prostatectomy patients in the proportion of men with elevated levels of depression, anxiety, or distress. - Among 177 men who underwent radical prostatectomy and has complete follow-up moderate or higher levels of depression or anxiety appeared to be associated with post-treatment SF (sexual function) and bother, while elevated levels of distress were associated with post-treatment urinary function. |
| Bellardita [63]  2013  Italy  AS | Single cohort | To identify factors associated with poor quality of life during AS | 103 patients with a mean age of 67 years.  76 (73.7%) had a partner, 58 (56.3%) had primary, middle, and vocation school education, 74 (71.8%) had no financial worries, and 55 (53.3%) are not physically active | - Lack of partner (OR: 0.08; p=0.009) and impaired mental health (OR: 1.2, P=0.1) were associated with low health-related quality of life (p=0.006; are under the curve [AUC]: 0.72). - The maladaptive adjustment to cancer (p=0.047; AUC: 0.60) could be predicted by recent diagnosis (OR: 3.3; p=0.072). - Poor global quality of life (overall p=0.02; AUC: 0.85) was predicted by impaired mental health (OR: 1.16; P=0.070) and time from diagnosis to enrolment in AS <5 months (OR: 5.52; p=0.009). - Influence of different physicians on the choice of AS (OR: 0.17; p=0.044), presence of a partner (OR: 0.22; p=0.065), and diagnostic biopsy with >18 core specimens (OR: 0.89; p=0.029) were predictors of better quality of life. |
| Mroz [64]  2013  Canada  AS | Qualitative  (interviews) | To describe connections between masculinities and patient perspective of AS related communication with male physicians | 25 men on AS  14 (56%) men were between the ages of 65-70, 9 (36%) had a university degree, 19 (76%) were married/cohabiting, 12 (48%) were retired, and 23 (92%) were Caucasian. | - Patient-physician communication of diagnosis, treatment options, and specificities of AS featured prominently within participants’ interviews. - Most patients reported brevity in patient-physician communication amid accepting the physician’s communication style and recommendations. - Participants’ accounts suggested “therapeutic” communications as contingent on positioning physicians as authoritative and assigning them control, while patients were stoic in accepting AS. - However, some participants reported being confused about their diagnosis details and specific AS protocols, and resisted traditional physician-patient hierarchies, desiring more collaborative decision-making processes. - In this regard communications emerged as “threat,” in that participants lacked and/or lost confidence in their physician and were uncertain and anxious about the legitimacy of AS. - These findings were interpreted within a gender framework, showing how patients drew on an array of masculine ideals, including stoicism, denying illness, and respecting expertise in different ways |
| Loeb [65]  2013  USA  AS | Single cohort | Examine contemporary trends in AS and watchful waiting in the nationwide Swedish prostate cancer registry. Also examined factors associated with selection of deferred management, which might provide insight into the rational diffusion of this important management strategy. | 57,713 men with low or intermediate risk prostate cancer  13,272 (46%) men with low risk and 8,695 (30%) with intermediate risk prostate cancer chose deferred treatment.  Mean age of low risk was 65 years with a range of 60 to 70 years  Mean age of intermediate risk was 69 years with a range of 63 to 75 years | - Since 2007, 59%, 41% and 16% of very low, low and intermediate risk prostate cancer, respectively, chose AS. - Age was by far the strongest determinant of deferred treatment. - Education, marital status and comorbidity were significantly but weakly associated with deferring treatment. - For very low risk patients the highest likelihood of AS was observed for men aged 6 to 69 years at diagnosis (OR 3.40, 95% CI 2.11 to 5.49). |
| Xu [66]  2012  USA  WW/AS | Qualitative  (interviews) | To describe prostate cancer treatment decision making, focusing on knowledge and attitudes toward observation, Watchful Waiting or AS, and reasoning for not choosing them | 21 men with localized prostate cancer  14 black; 7 white | - All cancers were detected by prostate-specific antigen screening; 14 men had low-risk disease. - 19 chose surgery or radiation treatment. - The majority wanted to “get rid of” or “cure” the cancer by undergoing aggressive therapy, even with awareness of the potential for significant side effects. - Most men seemed unaware of the uncertainty/controversies that aggressive treatment may not cure their cancer or improve their survival. - Limited knowledge about Watchful waiting/AS was common, and few remembered Watchful waiting/AS being presented as a viable option. - Many men perceived it as “doing nothing.” - Some men, who initially were inclined toward Watchful waiting/AS, yielded to pressure from family, physicians, or both to choose aggressive treatment. - Lack of physician support was a significant barrier to Watchful waiting/AS. |
| Seiler [67]  2012  Switzerland  AS | Single cohort | To assess anxiety levels and health related quality of life in both patients with prostate cancer on AS and their partners | 133 couples (response rate of 46.9%)  The mean age was 66.2 years in partners and 69.3 in men. | - At the time quartiles, partners had anxiety scores of 5.5, 4.6, 5.4, and 5.6. - Scores in men were statistically significantly lower: 3.9 (p=0.05), 2.0 (p<0.001), 3.3 (p=0.002), and 3.3 (p=0.02), respectively. - The partners’ scores were still well below 7 (ie, normal). - Prostate-specific anxiety scores were below the clinical threshold as well: 15.5, 9.5, 6.5, and 9.0, respectively. |
| Goh [68]  2012  USA  AS | Qualitative  (interviews) | To understand the factors associated with decision-making | 34 individuals on AS for prostate cancer  Overall, most of the respondents were non-Hispanic white (84.9%), involved in a primary relationship (94.1%), and had a mean age of 63.1 years. | - Primary analyses focused on the decisional satisfaction and conflict measures, as the decisional regret measure showed poor reliability (α < 0.7) in this sample - Four psychosocial measures showed strong associations across the decision-making subscales, including the Fife Constructed Meaning Scale (Pearson r > 0.26), Mishel Uncertainty in Illness Scale – Inconsistency (r>0.32), Mental Health Index-5 (r > 0.33), and Lepore self-efficacy for prostate symptom management scale (r > 0.33). - Individuals with higher self-efficacy for prostate cancer symptom management (p=0.02) and higher positive meaning for cancer (p=0.03) were less likely to express decision-making conflict as the result of uncertainty. - Individuals reporting higher positive meaning for cancer (p=0.01) and less uncertainty in illness attributed to inconsistency (p=0.02) were less likely to exhibit decision-making conflict related to the perceived effectiveness of treatment |
| Davison [69]  2012  Canada  AS | Single cohort | To assess information and decision making preferences of patients on AS, and the factors influencing their decision | 180/258 (69.8%) of patients on AS for <10years agreed to participate in this study  The mean age of all participants was 67.2 years, with 91 (50.6%) being between 61 to 70 years.  84 (46.7%) had university level education, 104 (57.8%) were retired, 144 (80%) were married/cohabitating, and 153 (85%) were Caucasian. | - 35% of patients reported assuming an active role in treatment decision making, 38% in a collaborative role, and 27% a passive role. - Results suggest that patients <60 years prefer to play an active role in treatment decision making whereas, men >70 years prefer to play a passive role. - Available treatment options, eating a ‘prostate friendly’ diet, and non-traditional therapies were identified as the top three information preferences. - Patients with higher levels of anxiety wanted access to more information compared to those with lower levels of anxiety. - The urologists’ recommendation was rated the most important factor influencing patients’ decisions to go on AS |
| Gorin [70]  2012  USA  AS | Questionnaire | To survey urologists regarding their knowledge, acceptance and practice of AS for low-risk prostate cancer | 425/4987 (9%) urologists successfully completed the survey  Of the 387 familiar with AS:  169 (44%) completed fellowship training, 105 (57%) speciality was oncology, 266 (69%) practiced in the United State, and 189 (49%) were community-based | - 387 (91%) of urologists were both familiar with AS and aware that AS differed from watchful waiting. - Of this group, 370 (96%) respondent felt AS was a reasonable management strategy, 95% of whom manage patients with this approach. - A minority of respondents (6%) felt that patients with a prostate specific antigen > 10ng ml-1 were eligible for AS. - Most participants (74%) felt that patients required a Gleason score ≤6. - There was little agreement on timing of follow-up biopsies. - Respondent who objected to AS were most commonly concerned with missing an opportunity for curative treatment (76%) and the risk of tumor undergrading (65%) |
| Vasarainen [71]  2012  Finland  AS | Single cohort | To analyse longitudinal changes in general, mental and physical health related quality of life and urinary and erectile function in patients with low-risk prostate cancer on AS | 124 patients  80/124 patients on AS were followed for a year  105/124 (85%) returned the baseline RAND-36 questionnaire  75/80 (94%) answered both the baseline and follow-up questionnaires  Of the 75 patients, 51 (67%) were married or living with a partner and the median age at diagnosis was 64 years old | - No differences existed in the health related quality of life main categories at the 1-year follow-up (mental and physical: p=0.142 and p=0.154, respectively). - When all the eight dimensions were analysed separately, the physical role showed statistically significant improvement from a mean of 81 to a mean of 89 (p=0.010). - No clinically significant correlations appeared between health related quality of life and age, diagnostic prostate-specific antigen, free prostate-specific antigen or prostate-specific antigen change during follow-up at any of the time points; in regression analysis, health related quality of life was not predictable by any of the variables available at diagnosis or during follow-up. - No statistically significant changes occurred in urinary function as analysed by the International Prostate Symptom Score (p=0.121) or in erectile function by the International Index of Erectile Function-5 questionnaire (p=0.583). - Compared with an age-stratified Finnish general male population, patients with PC on AS had a significantly better general mental and physical health related quality of life at diagnosis and after 1 year of follow-up (p<0.05). |
| Sidana [72]  2012  USA  AS | Comparative cohort | To provide insight into information sources consulted and factors dictating treatment decision-making in young prostate cancer patients | 1511 men under 50 years old diagnosed with Gleason score 6 prostate cancer between 2001-2005  Current addresses were obtained for 986 (64%) patients. 493 (50%) responses were obtained.  397 (81.4%) chose surgery with a mean age of 45.7 years, 350 (90%) were white and 237 (59.9%) had grad school education.  52 (10.7%) chose radiation with a mean age of 46.6 years, 42 (80.8%) were white and 30 (58.8%) had grad school education.  26 (5.3%) chose AS with a mean ago of 46.6 years, 25 (96.2%) were white and 18 (69.2%) had grad school education.  13 (2.7%) chose other treatments with a mean age of 42.9 years, 12 (92.3%) were white and 8 (61.5%) had grad school education. | - Participants with at least some college education (p=0.003) or annual income >$100,000 (p=0.003) were more likely to consult three or more doctors. - Amongst all treatments, ‘doctor’s recommendation’ was the most influential information source, although relatively less important in the AS group. - Internet was the second most frequent information source. - Participants with higher education (p=0.0003) and higher income (p=0.002) considered sexual function more important while making a treatment choice. - Only 2% of men preferred a passive role in the decision-making. - Informed decision-making was preferred more by patients who chose radiation and AS while shared decision-making was preferred more by surgery patients (p<0.05). - The majority (89%) of the respondent did not regret their decision. - No difference in satisfaction levels was found between different treatment modalities - Among demographic attributes, only marital status was significantly associated with treatment, with more married men choosing surgery or radiation than AS (p<0.002). - Physicians were cited as an information source somewhat more frequently among whites than non-whites (97% vs. 90%, p=0.04). - Among patients whose physicians endorsed a single treatment, 91% received a recommendation for surgery and while only 4% were recommended radiation. - Among patients who chose surgery, 79% said that the doctors recommended against AS, while only 412% of patients who chose AS said that the doctors recommended against AS (p<0.0001). |
| Kazer [73]  2011  USA  AS | Single cohort | To provides preliminary data on an Internet intervention that incorporates cognitive reframing and self-management strategies to help older men undergoing AS self-manage disease-related issues and improve quality of life | 9 patients undergoing AS  Mean age was 72 years with a range of 66 to 79 years.  All patients were Caucasian and 7 were married.  3 had some college education, 3 were college graduates, and 3 had graduate or professional school educations | - The results revealed change between baseline (time 1) and intervention completion (time 2) in the majority of variables, with a return toward baseline after the intervention (time 3; 5 weeks after intervention completion). - 10 of the 12 measures of intervention acceptability were met. - The results showed positive trends in the impact of the intervention and good overall acceptability. - The data revealed that men viewed the Web pages 2 to 40 times, with an average of 20 page views per participant. - Results revealed significant relationships between the number of Web page views and two quality of life domains: role function related to emotional health (r=0.88, p=0.02) and social function (r=0.88, p=0.02) in T3. |
| Kazer [74]  2011  USA  AS | Qualitative  (focus groups) | To determine the psychosocial educational needs of men undergoing AS for prostate cancer | 7 participants over the age of 65 diagnosed with prostate cancer and currently undergoing AS  Participants were Caucasian and ranged in age from 65 to 79 years with a mean age of 70. | - The results revealed that participants of these focus groups frequently turned to the Internet for information related to prostate cancer and AS. - As the data revealed that men may not discuss their concerns with other in traditional support group structures or with their spouse and children, the Internet could provide this population with an acceptable method of receiving education and support. - The study also found frequently occurring concerns regarding disease surveillance for which participants continued to actively monitor their cancer and seek second opinions. - More education related to prostate cancer may play a substantial role in managing uncertainty related to the disease. - Participants in this study also made a number of lifestyle changes upon diagnosis with prostate cancer. |
| Xu [75]  2011  USA  WW | Qualitative  (interviews) | To explore how black and white American men made their treatment decisions | 21 men (14 black and 7 white) recently diagnosed with localized prostate cancer | - Physician recommendation was very important in the treatment decision, but patient self-perception/values and attitudes/beliefs about prostate cancer were also influential. - Patients who chose surgery believed it offered the best chance of cure and were more concerned that the cancer might spread if not surgically removed. - Patients who chose radiation therapy believed it offered equal efficacy of cure but fewer side effects than surgery. - Fear of future consequences was the most common reason to reject watchful waiting. - Anecdotal experiences of family and friends were also important, especially in deciding “what not to do.” - The new technology of robotic assisted prostatectomy provided optimism for men who wanted surgery but feared morbidity associated with traditional open surgery. - Few men seemed aware that treatment did not guarantee improved survival. |
| Davison [76]  2011  Canada  AS | Single cohort | To examine the decision-making processes of men on AS and to identify the resources that men want to access to make, support, and sustain them while on AS | 73 men on AS for <10years  64.49 years was the average age and 37 (50.7%) were between the ages of 61 to 70 years old.  36 (49.4%) had university as educational attainment, 60 (82.2%) were married, 45 (61.6%) were retired, and 63 (86.3%) were Caucasian. | - 27% of the 73 men reported assuming an active role in treatment decision-making with their urologist, 41% shared role and 32% a passive role. - 82% of men reported being comfortable and 90% being satisfied with their decision to be on AS. - 55% reported not being anxious about the cancer progressing while on AS. - Urologist’s opinion, current age, and impact of treatment on urinary function were main factors influencing treatment decision. - Compared with participants (n=40) who reported ‘none’ to ‘very little’ levels of anxiety over being on AS, participants (n=33) who reported having levels of anxiety ranging from ‘somewhat’ to ‘a great deal’ reported that talking to other men on AS (t=3.33, p=0.001), receiving information about future treatment options (t=2.18, p=0.03), having support group for men on AS (t=2.38, p=0.02), and joining a web-based anonymous support group for only men on AS (t=2.44, p=0.01) as being important. - AS was the treatment recommended by most (84%) urologists. - Most (>75%) of the men reported that the decision to go on AS was similar to other life decisions; the decision was not difficult; and they were comfortable being on AS. - Compared with participants (n=40) who reported ‘none’ to ‘very little’ levels of anxiety over being on AS, participants (n=33) who reported having levels of anxiety ranging from ‘somewhat’ to ‘a great deal’ reported that current age (t=4.51, p≤0.001), advice from my family doctor (t=2.73, p=0.008) and current health (t=2.05, p=0.04) were significantly more important. |
| Anandadas [77]  2011  UK  AS | Comparative cohort | To identify the reasons for patients with localised prostate cancer choosing between treatments and the relationship of procedure type to patient satisfaction post-treatment | Of 768 patients, 305 (40%) chose surgery, 237 (31%) conformal beam radiotherapy (CRT), 165 (21%) brachytherapy and 61 (8%) AS (AS).  Median age (and range) were 62.5 (44 to 75), 64.3 (51 to 76), 62 (49 to 74) and 64.8 (44 to 74) years, respectively. | - 60% of men who opted for radical prostatectomy were motivated by the need for physical removal of the cancer. - Conformal radiotherapy was mainly chosen by patients who feared other treatments (n=63, 27%). Most men chose brachytherapy because it was more convenient for their lifestyle (n=64, 39%). - AS was chosen by patients for more varied reasons. Post-treatment satisfaction was assessed in a subgroup who took part in the quality of life aspect of this study. - Of the respondents to the questionnaire, 212 (87.6%) stated that they were satisfied/extremely satisfied with their choice and 171 (92.9%) indicated they would choose the same treatment again. - Those that chose AS suggested they ‘didn’t want active or invasive treatment.’ - The modest increase in AS during the study reflect the increase popularity of less invasive treatment approaches and AS amongst patients and doctors in recent years - Of the 768 patients 24.2% chose ‘physical removal of cancer’ as their reason for their treatment choice. |
| Gorin [78]  2011  USA  AS | Single cohort | To learn from patients their rationale for enrollment in AS for low-risk prostate cancer as an alternative to primary treatment | The survey was returned by 105 (57%) of 185 patients.  The mean age of the respondents was 65.5 years. | - AS was offered to 38 (36%) of 105 patients by the physician who had made the initial diagnosis. - Patients most frequently reported physician influence as the greatest contributor to their decision to elect AS (73%). - Patients also cited concerns regarding the potential side effects of incontinence (48%) and erectile dysfunction (44%) associated with therapy as reasons for choosing AS - Statements receiving the greatest number of 1s included: “My doctors thought it was a reasonable alternative”; “I felt sure I could still be cured with treatment if my cancer progressed”; and “I have researched the alternative and this one seems the best for the type of prostate cancer I have.” |
| Ramsey [79]  2011  USA  AS | Questionnaire | To describe urologist recommendations for treatment among local-stage prostate cancer patients presenting for initial management consultations versus second opinions | Of the 238 eligible patients, 95 men presented for initial consultation, and 143 men presented for a second opinion  Of the 238 patients, 36% were <60 years old, 71% were white, 44% worked full-time and 27% had some college education. | - Urologists recommended 0.52 more treatments (standard error 0.19, p<0.001) during an initial consultation as opposed to a second opinion. - The proportion recommending surgery increased from 71 to 91% (initial consultation versus second opinion setting). - Among initial consultations, 59% had low-risk disease, and urologists’ recommendations included surgery (80%), external radiation (38%), brachytherapy (seeds) (52%), and AS (25%). - Of the 54% with low-risk disease in a second opinion consultation urologists’ recommendations included surgery (90%), external radiation (16%), brachytherapy (14%), and AS (16%). - Urologists were less likely to recommend AS. AS was not strongly recommended by either speciality |
| Ervik [80]  2010  Norway  AS | Qualitative  (interviews) | To illuminate men’s experiences living with localized or local advanced prostate cancer when curative treatment such as surgery or radiation therapy is not an option at the time of diagnosis | 10/21 (47%) men were included in the study.  7 were treated with endocrine therapy and 3 with AS  Age range of participants was 59083 years with a median of 71 years.  9 were married and 8 had the working status of pensioner | - Being diagnosed with prostate cancer was described as a shock, with different aspects of the illness revealed gradually. - The limited amount of time available for meeting with health care providers contributed to patients’ feelings of being left alone with difficulty getting information and help. - Sexual and urinary problems were perceived as a threat to their manhood. - Sexuality was an important subject, especially for the men around 60 years and those receiving endocrine therapies. - The spouses provided the closest everyday support. |
| van den Bergh [81]  2010  The Netherlands  AS  van den Bergh [82]  2010  The Netherlands  AS  van den Bergh [83]  2009  The Netherlands  AS | Single cohort | Exploring how anxiety and distress may be present and levels of knowledge of prostate cancer and the perception of AS in patients with low risk prostate cancer who are on AS and how this may be a reason to discontinue AS | 129/150 (86%) Dutch patients with prostate cancer on AS completed the first questionnaire  Median age was 64.6 years. 86 (67%) had low (primary, secondary) education, 76(60%) were employed, 119 (92%) were married/living together and 93 (73%) were sexually active. | - Significant but clinically irrelevant decreases were seen in mean scores of the State Trait Anxiety Inventory (p=0.016), Memorial Anxiety Scale for Prostate Cancer fear of progression subscale (p=0.005) and the self-estimated risk of progression (p=0.049). - Higher Eysenck Personality Questionnaire neuroticism score and an important role of the physician in the treatment decision had additionally unfavourable effects. - Good physical health, palpable disease and older age had favourable effects. - Patients with low risk prostate cancer who chose AS show favourably low anxiety and distress from the time of diagnosis up to 9 months later. - Men with low neurotic personality and good physical health scores seem to psychologically perform best during AS - The most frequently reported advantage and disadvantage of AS were the delay of side effects and the risk of disease progression, respectively. - Significantly more men failed to provide any disadvantage than any advantage (p<0.01). - Specific negative experiences included the feeling of losing control over treatment decisions, distress at follow-up visits, and the desire for a more active participation in disease management. - No conceptually wrong understandings or expectations of AS were identified - The following associations emerged: a perceived important role of the physician in shared decision-making was associated with higher decisional conflict, better physical health was associated with lower depression, neurotic personality was associated with higher depression and with generic and prostate-specific anxiety, and higher prostate-specific antigen level was associated with higher prostate cancer-specific anxiety. |
| Couper [84]  2009  Australia  WW | Comparative cohort | To assess the psychological impact of the different treatments for localised prostate cancer | 211 patients with prostate cancer attending clinics in public hospitals and private practices in metropolitan Melbourne between 1 April 2001 and 30 December 2005  Mean age was 66.15 years (range, 43 to 92), 78.3% were married and 63.2% were retired/ unemployed/ pensioner  193 completed the time 1 questionnaires (38 radical prostatectomy, 56 hormone therapy, 38 other early treatment and 61 watchful waiting)  172 completed the time 2 questionnaires (33 radical prostatectomy, 51 hormone therapy, 33 other early treatment and 55 watchful waiting) | - Patients were sorted into three active treatment groups: radical prostatectomy, hormone or other early treatment including radiation therapies. - At Time 1, the three active treatment groups all reported greater dysfunction in work role and daily activities compared with the watchful waiting group. - The radical prostatectomy group also reported worse social and emotional role functioning, while the hormone therapy and other early treatment groups reported poorer vitality levels. - The hormone therapy group reported significantly higher depression scores. - Patients who elected to undergo watchful waiting were treated as a naturalistic control group. - All groups reported greater dysfunction in work role and daily activities compared with the watchful waiting. - At time 2, the radical prostatectomy and other early treatment groups did not differ from the watchful waiting group on either health related quality of life or psychological status. - By contrast, the hormone therapy group reported significantly worse health related quality of life (physical functioning, role-physical and vitality domains) and greater psychological distress compared with the watchful waiting group. |
| Oliffe [85]  2009  Canada  AS | Qualitative  (interviews) | To describe the range of men’s self-management strategies used to overcome AS -related uncertainty | 25 men on AS  The participants ranged in age from 48 to 77 years (M=68), and most were married White men (n=19) who had completed postsecondary education (n=18). | - The study findings reveal 2 strategies: first, positioning prostate cancer as benign through stoicism and solitary discourses were common to men intent on “living a normal life.” - Second, men committed to “doing something extra” complemented AS protocols, and often collaborated with their wives to focus on diet as an adjunct therapy. - Despite trusting their doctors, and being relieved the have avoided treatments and their potential side effects, all the men we spoke with reported some uncertainty about being on AS. - Most men were concerned about their mortality and the potential for the cancer to spread beyond the prostate gland, rendering them ineligible for curative treatments - The potential imminent need for treatment created uncertainty about how men might cope with treatment-induced morbidities (i.e. exacerbated erectile dysfunction and/or urinary incontinence) - Men’s uncertainty was time sensitive and peaked leading up to the schedules AS appointments and impending prostate specific antigen and/or TRUS-Bx results |
| Davison [86]  2009  Canada  AS | Qualitative  (interviews) | To identify and describe decision-making influences on men who decide to manage their low-risk prostate cancer with AS | 25 patients diagnosed with low-risk prostate cancer on AS  The mean age of men in the study was 66 years. 17 (68%) were between the ages of 61 to 70 years.  9 (36%) men had an undergraduate university degree, 19 (76%) were married or cohabiting, 12 (48%) were retired and 23 (92%) were Caucasian. | - The specialists' description of the prostate cancer was the most influential factor on men choosing AS. - Patients did not consider their prostate cancer to be life threatening and, in general, were relieved that not treatment was required. - Avoiding treatment-related suffering and physical dysfunction and side effects such as impotence and incontinence was cited as the major reason to delay treatment. - Few men actively sought treatment or health-promotion information following their treatment decision. - Female partners played a supportive role in the decision. - The need for active treatment if the cancer progressed with acknowledged. - Patients were hopeful that new treatments would be available when and if they needed them. - Being older and having comorbidities did not preclude the desire for future active treatment. - Patients carried on with their lives as usual and did not report having any major distress related to being on AS. - Biopsies were described as a painful necessity of the treatment plan but “better than having surgery.” |
| Arrendondo [87]  2008  USA  WW | Single cohort | Report on health related quality of life in men with localized prostate cancer who selected watchful waiting | 310 men diagnosed with prostate cancer from 1990 to 2001 within Cancer of the Prostate Strategic Urological Research Endeavor who chose watchful waiting  The mean age of the entire cohort was 74.7 years. 185 (59.7%) were 75 years or older. 272 (87.7%) were white, 235 (75.8%) were in a significant relationship, and 157 (50.7%) had high school or less as their highest education | - Urinary function shows a mild age effect and a near 0 decrease in time trend after diagnosis. The trend observed with urinary function after the diagnosis of prostate cancer is consistent with the effect of aging. The age effect pattern is similar for urinary bother although not statistically significant. - Sexual function and sexual bother; after diagnosis is greater than expected from aging alone but the difference is only statistically significant for sexual function (p=0.0006). - Physical function and role physical showed a statistically significant age effect and time trend while vitality, social function, bodily pain and general health demonstrated a statistically significant time trend. - 2 domains to be statistically significant at the p=0.01 level (urinary function p=0.013 and urinary bother p=0.028). - Patients who choose watchful waiting can expect an extremely small decrease in general health related quality of life over time. - Men who choose WW usually have a lower risk disease profile, more comorbidities and are older compared to men who seek active treatment. - Men who chose watchful waiting reported better or similar health related quality of life scores for 7 of the 16 health related quality of life domains compared with men who did not have prostate cancer. - There were 3 domains – urinary function, urinary bother and sexual function – in which the watchful waiting group reported a lower health related quality of life score compared to men without prostate cancer. - Significant decreases with time were observed in 7 domains of the RAND 36-Item Health Survey and 4 of the UCLA Prostate Cancer Index scales. |
| Isebaert [88]  2008  Belgium  WW | Single cohort | To determine the impact of a decision aid for patients with localized prostate cancer on the consultation and the decision-making process. Specifically, they evaluated the positive and negative aspects associated with the use of the decision aid | 50 patients with newly diagnosed localized prostate cancer were interviewed once  The mean age was 71 years, 13 (26%) had an advanced (until 19^th^ year) education level, 40 (80%) were married, and 41 (82%) were living with their partner.  11 physicians were interviewed; 3 radiation oncologists and 8 urologists. | - The patients became more active partners in the decision-making process: they were better prepared for the consultation, asked more direct information, and were able to make a more deliberative choice. - Generally, the use of the decision aid improved the quality of the consultation and resulted in a treatment decision agreed upon by both parties. - Sometimes the consultation turned out to be more time-consuming. - The decision aid did not only improve the patient-physician interaction but also helped patients to discuss the disease with their partner and family members - The majority of patients (45/50) felt that the decision aid contains an adequate amount of information and that the different treatment options are equally presented (46/50). - Especially the section on the advantages, the disadvantages and the side effects of each treatment option was highly appreciated (50/50). - In 76/93 interviews the physicians believe that the decision aid influenced the consultation and the decision-making process positively. - Remarkably, the physicians thought more often than the patients themselves that there was too much information in the decision aid and/or that this information was too difficult (p=0.001; Fisher exact test). |
| Sommers [89]  2008  USA  WW | Comparative cohort | This study examined determinants of patients’ preferences and/or other factors predicting treatment choices | 167 patients with newly diagnosed localized prostate cancer  Average age was 62 years old. 88% were white non-Hispanic, 45/5% had college education, and 64.7% had an income >$50,000. | - Patient preferences were affected by a range of behavioural, demographic, and health factors. - For example, sexually active men reported significantly lower quality-adjusted life years for living with erectile dysfunction, and men with family members who died of cancer reported lower quality-adjusted life years for metastatic disease. - The most common choice was radical prostatectomy (37%) 4.2% chose watchful waiting. - Watchful waiting was more commonly chosen by men expressing a desire to avoid side effects. - The main decision factor was physician's advice. - The strongest predictor of treatment was the type of physician seen (radiation oncology vs urology) at the time of the survey. - Age and tumor grade also were found to be strongly predictive of treatment. - In general, quality-adjusted life years were not found to predict treatment choice. - *Radical prostatectomy.* Younger men were more likely to choose radical prostatectomy. Patients experiencing frequent thoughts of death also were more likely to choose radical prostatectomy. radical prostatectomy was less likely to be chosen by men who reported “avoiding side effects” as their top priority. *External beam radiation.* Men with high- or medium-risk tumors were significantly more likely than those with low-risk tumors to choose external beam radiation. *Brachytherapy.* Older men were more likely to choose brachytherapy, as were men with low-risk tumors. Men who prioritized “avoiding side effects” were more likely to select brachytherapy.  *Hormonal therapy*. Hormone therapy was more common in patients with high- and medium-risk tumors. Notably, hormone therapy was used as monotherapy for just 1 patient in our sample, and otherwise was always an adjuvant to external beam radiation or brachytherapy. |
| Taussky [90]  2008  Canada  WW | Comparative cohort | To analyse factors influencing treatment decision in patients diagnosed with low-risk prostate cancer who were referred to a brachytherapy clinic and had to choose from four treatment options: expectant management (watchful waiting), radical prostatectomy, external beam radiation therapy, and permanent seed brachytherapy | 110 patients with low risk prostate cancer.  52.7% were between the ages of 60 to 70 years old.  53 patients (48.2%) chose PB (permanent seed brachytherapy), 33 patients (31.8%) chose expectant management, 12 patients (10.9%) chose external beam radiation therapy, and 10 patients (9.1%) chose radical prostatectomy. | - Patients who chose brachytherapy were significantly younger than those who chose external beam radiation therapy (p=0.011). - Patients living further away from the hospital than the median distance of 19.85 miles were more likely to choose brachytherapy than expectant management (p=0.017). - Patients with urinary voiding symptoms above the median of 7 were more likely to choose external beam radiation therapy over brachytherapy, probably partly due to a selection bias, since patients with more urinary symptoms were discouraged from undergoing brachytherapy. - Compared to patients with an International Prostate Symptom Score of 7 or less, those with an International Prostate Symptom Score greater than 7 were 71% less likely to choose brachytherapy. |
| Hegarty [91]  2008  USA  AS | Comparative cohort | To enhance the understanding of the experience of AS for prostate cancer among Irish and American men by measuring quality of life and levels of uncertainty among men over the age of 65 in receipt of the AS management option for prostate cancer | 29 men undergoing AS over the age of 65  In southern Ireland (n=10) and the United States (n=19).  Mean age of the United States group was 76 years, with a range of 65 to 85 years. 47% were married, had an annual income of <$20,000 and had a high school education.  Mean age of the Ireland group was 76.5 years, with a range of 72 to 85 years. 67% were married, 50% has an annual income of <$20,000 and 60% had a high school education | - Men undergoing AS in the United States have slightly higher levels of uncertainty. - Primary appraisal, opportunity, and danger appraisal were consistent between samples from both countries. - Total affective and health-related quality of life scores were similar among AS participants in both countries, but subscale scores identified both similarities and differences. - Irish men had lower mean role and social function that United States men, and higher general health and energy. - Irish men reported more urine bother and less sexual bother than United States men. |
| Burnet [92]  2007  UK  AS | Comparative cohort | To investigate the prevalence of anxiety and depression in patients with localised prostate cancer managed by AS, compared with those receiving immediate treatment | 329 men with localized disease completed the HADS  100 were on AS  81 receiving radical treatment  148 previously received radical radiotherapy  AS group: 95 (95%) White British, 78(78%) married/with partner, 35(35%) had secondary education and 76(76%) were employed.  Radical treatment group: 75 (92.6%) White British, 71 (87.7%) married/with partner, 27 (33.3%) had secondary education, and 67 (82.7%) were retired.  After treatment group: 137 (92.6%) White British, 126 (85.1%) married/with partner, 45 (30.4%) had university education, and 120 (81.1%) were retired. | - 16% (51/329) of patients met the HADS criteria for anxiety and 6% (20/329) for depression. - Analyses indicated that higher anxiety scores were significantly associated with younger age (p<0.01) and a longer interval since diagnosis (p<0.01), but not with management by AS (p=0.38). - Higher depression scores were significantly associated with a longer interval since diagnosis (p<0.05), but not with management by AS (p=0.83). - AS for managing localized prostate cancer was not associated with greater psychological distress than more immediate treatment for prostate cancer. - Frequency analyses indicated a low prevalence of anxiety and depression, with 21 and 4/100 respectively on AS scoring above the threshold. |
| Katz [93]  2007  USA  WW | Comparative cohort | To investigate the long-term changes in health related quality of life, continence, and sexual function after curative therapy and watchful waiting for prostate cancer | 61 patients  20 chose watchful waiting (33%)  41 chose curative therapy (67%)  The 62% were white and 38% African American with a mean age of 65.8 years. | - The average follow-up for the curative therapy patients was 24.3 months. - The pre-treatment incontinence score was 0.38 and the health related quality of life burden score was 16.3 (curative therapy versus watchful waiting, p=0.55). - On follow-up, 23 (56%) of 41 curative therapy patients were continent. - The health related quality of life burden score after treatment was similar between the curative therapy and watchful waiting patients (20.4 versus 18.4, p=0.45). - The continent patients had a significantly lower health related quality of life burden compared with the post- curative therapy incontinent patients (17.41 versus 24.2, p=0.02). - The health related quality of life burden score after treatment for curative therapy patients who maintained sexual activity and curative therapy patients who lost their sexual activity was similar (P=0.28). - The watchful waiting patients maintain their health related quality of life. - 75% of the watchful waiting patients who were sexually active before treatment reported being sexually active at follow-up. |
| Clarke [94]  2007  UK  AS | Questionnaire | To assess which clinical parameters consultant urologists use to recommend treatment for early prostate cancer | 30 consultant urologists reviewed 70 paper representations of patients with prostate cancer. | - Consultants varied in the treatments that they recommended. - An average of only 3 of the possible 9 cues was used to formulate decisions. - Prostate specific antigen and predicted 10-year survival probability were most commonly used for recommending all 3 treatment options. - Patient choice, predicted life expectancy, rectal examination and were all used infrequently. - Consultants were inconsistent in an average of 31.4% of judgments when repeat cases were analyzed with the greatest inconsistency observed when recommending radiotherapy with or without hormones and the least inconsistency when recommending radical prostatectomy. - For AS consultants most commonly used predicted 10-year survival probability, magnetic resonance imaging/laparoscopy stage and prostate specific antigen when recommending this treatment, as indicated by a significant correlation with fractional score in 23, 16 and 10, respectively (p<0.01). |
| Bailey Jr [95]  2007  USA  WW | Qualitative (interviews) | This study explores the problems and uncertainties of older men, with prostate cancer, who have undergone watchful waiting and the strategies they use to manage their concerns | 10 men who were undergoing watchful waiting for prostate cancer  2 of the men were African-American and 8 were Caucasian; their ages ranged from 64 to 88 years.  The men had been living with prostate cancer from 4 to 12 months and had elected for watchful waiting in conjunction with their healthcare provider. | - Domains of uncertainty, appraisal of danger and appraisal of opportunity were identified and each was supported by participant’s experience. - A defining feature of participant’s uncertainty was the fact that prostate cancer offered few signals for them as they tried to monitor the progression of their disease. Men who had no physical discomfort found it hard to believe the cancer existed, - Some men in the study worried that getting a second opinion would add to their conflict about the decision to watch and wait. - Several of the men viewed their decision to watch and wait as an opportunity to successfully manage their uncertainty through work, self-care, keeping options open, and the use of alternative medications and prayer. |
| Joseph [96]  2006  USA  WW | Comparative cohort | To assess perception of stress and quality of life in a sample of prostate cancer survivors | The respondents (n=136) ranged in age from 39 to 92 years, with a mean of 70.5 years. The majority were married/living with a spouse (69%, n=94) and retired (86%, n=117).62%, n=84 were Caucasian/European Americans.  Treatment options included surgical intervention/prostatectomy (50%, n=68), radiation (25%, n=34), a combination of radiation and hormone therapy (10, n=13), watchful waiting (7%, n=10), and hormone therapy (6%, n=8). | - The findings revealed low levels of stress with marginal reports of quality of life. - Significant differences were found in organ-specific functioning (p<0.001), with respondents indicating that they were experiencing a disproportionately higher rate of sexual problems (sexual performance and sexual satisfaction) compared to bowel and bladder problems. - These complaints were highest among patients who had undergone prostatectomy and lowest among patients who had selected watchful waiting. - No association was found between stress and quality of life, but significantly higher rates of stress were reported by patients who felt they had not received sufficient information before treatment (p<0.05). |

WW watchful waiting; AS active surveillance
